# Non-Hodgkin Lymphoma and Occupational Exposure to Agricultural Pesticide Chemical Groups and Active Ingredients: A Systematic Review and Meta-Analysis

**DOI:** 10.3390/ijerph110404449

**Published:** 2014-04-23

**Authors:** Leah Schinasi, Maria E. Leon

**Affiliations:** Section of Environment and Radiation, International Agency for Research on Cancer 150, Cours Albert Thomas, 69372 Lyon Cedex 08, France; E-Mail: leonrouxm@iarc.fr

**Keywords:** pesticides, insecticides, herbicides, fungicides, lymphoma, non-Hodgkin, occupational, agricultural

## Abstract

This paper describes results from a systematic review and a series of meta-analyses of nearly three decades worth of epidemiologic research on the relationship between non-Hodgkin lymphoma (NHL) and occupational exposure to agricultural pesticide active ingredients and chemical groups. Estimates of associations of NHL with 21 pesticide chemical groups and 80 active ingredients were extracted from 44 papers, all of which reported results from analyses of studies conducted in high-income countries. Random effects meta-analyses showed that phenoxy herbicides, carbamate insecticides, organophosphorus insecticides and the active ingredient lindane, an organochlorine insecticide, were positively associated with NHL. In a handful of papers, associations between pesticides and NHL subtypes were reported; B cell lymphoma was positively associated with phenoxy herbicides and the organophosphorus herbicide glyphosate. Diffuse large B-cell lymphoma was positively associated with phenoxy herbicide exposure. Despite compelling evidence that NHL is associated with certain chemicals, this review indicates the need for investigations of a larger variety of pesticides in more geographic areas, especially in low- and middle-income countries, which, despite producing a large portion of the world’s agriculture, were missing in the literature that were reviewed.

## 1. Introduction

Striking increases in the incidence of non-Hodgkin lymphoma (NHL) cancer have occurred in the last 30 years [1,2], and interest in identifying environmental and occupational exposures associated with this cancer has accompanied this trend. Several environmental exposures have been proposed and investigated as potentially important—pesticides, dioxins, solvents, oils, and viruses, among others [3,4]. Farmers experience low overall mortality but high rates of some cancers; this suggests that some or several agricultural exposures may be key determinants [5,6]. Indeed, positive associations between NHL and farm related exposures, including pesticides, fertilizers, chemicals, animals, viruses, and endotoxin, have been observed previously [3,5,7]. However, the wide variety of chemical and microbial exposures that occur simultaneously in agricultural production makes disentangling the effects of these factors challenging. Of the many exposures experienced in farm settings, pesticides have drawn particular attention, especially since the increased incidence of NHL in the mid- to late-1900s followed widespread use of synthetic organic pesticides [4]. Also, several epidemiologic studies have reported positive associations between NHL and pesticide exposure in occupational manufacturing settings [8,9]. 

The United States Environmental Protection Agency defines pesticides as substances intended to prevent, destroy, repel, or mitigate a pest [10]. Within this broad category, pesticides are often grouped according to the type of pests that they control; for example, fungicides are used to kill fungi, insecticides to kill insects, and herbicides to kill weeds and plants. In addition to function, pesticides vary in terms of structure, and they are sometimes grouped according to chemical relationships. Furthermore, applicators often use a variety of pesticides simultaneously. These characteristics make designing and conducting epidemiologic studies of their health effects both challenging and expensive. 

Because pesticides are thought to have different toxicologic and immunologic effects, identifying the chemicals and chemical groups that are most dangerous to humans and non-target living organisms is important [11]. From a research perspective, the decision about what chemicals to investigate has implications for disease prevention, and it impacts the information that is available to policy makers and the public. 

These challenges and needs motivated us to systematically review the published epidemiologic literature of relationships of NHL with occupational exposures to agricultural pesticide chemical groups and active ingredients. The primary objectives of this paper were to investigate the depth of the literature on the relationship between specific pesticide chemicals and NHL, to identify gaps in this area of research, and to elucidate pesticide chemical groups and active ingredients that have shown particularly strong relationships with NHL. To help us to achieve these objectives, we conducted a series of meta-analyses of associations of individual pesticide chemicals with NHL.

## 2. Methods

### 2.1. Article Identification

We performed a search of literature on associations between occupational pesticide exposure and NHL. We restricted our search to articles published since 1980. This time period is consistent with that used in previous meta-analyses of farming exposures [5], and it captured the epidemiologic literature that has not been reviewed by early IARC monograph evaluations of pesticides [12]. The search used combinations of the following words: occupational exposure, pesticides, insecticides, herbicides, fungicides, neoplasms, cancer, lymphomas, non-Hodgkin lymphoma, cancer mortality, agricultural workers’ diseases/chemically induced, and humans. We entered combinations of these terms into PubMed and Web of Science. Details of the search are given in Supplementary file S1. 

### 2.2. Article Selection

To identify eligible studies, we reviewed the titles and abstracts of papers. When it was unclear from the abstract and title whether the paper fit these criteria, the full text of the paper was reviewed. We included estimates from papers with the following characteristics:

(1) Written and published in English;
(2) Reported results of analyses of case control or cohort epidemiology studies;
(3) Reported results of studies that used interviews, questionnaires, and/or exposure matrices to assess exposure;
(4) Reported associations of NHL with occupational, agricultural pesticide exposures;
(5) Reported quantitative associations of NHL overall and/or NHL subtypes with specific individual active ingredients or chemical groups.

We excluded papers with the following characteristics:

(1) Written in a language other than English;
(2) Did not report on associations with NHL;
(3) Were a commentary, letter to the editor, or monograph;
(4) Did not report associations with individual pesticide active ingredients or chemical groups; we excluded papers that reported associations with only the broadly defined categories of pesticide, insecticide, herbicide, or fungicide;
(5) Reported results of analyses of ecologic studies;
(6) Reported results of analyses of data from studies that were not case control or cohort in design;
(7) The exposure definition/classification was ambiguous;
(8) The exposure route was not occupational;
(9) The exposure route was not agricultural;
(10) Reported only associations within unique subpopulations (e.g., HIV positive patients);
(11) Reported analyses of manufacturing cohorts;
(12) Reported associations with NHL as a second primary;
(13) Reported results of studies in which exposure was assessed using biological markers.

### 2.3. Data Extraction

We extracted the following information from the full text of each eligible paper:

- author;
- publication year;
- study location;
- study design (case-control or cohort);
- source population for the controls in case-control studies;
- whether case-control studies were matched, and if so, the matching factors;
- diagnosis period if a case-control study or cancer follow-up period if a cohort study;
- number of cohort participants or number of cases and controls;
- cancer definition or ICD codes used to identify the cancers;
- method of assessing exposure;
- exposure metrics and definitions;
- referent categories used in the analysis;
- active ingredient(s) and/or chemical group(s) studied;
- covariates entered into the model to adjust for confounding;
- type of effect estimate reported;
- number of exposed participants;
- effect estimates and confidence interval limits; and
- gender restrictions, if any.

We also identified papers that were related to each other (e.g., pooled analyses that used data that were analyzed and reported on previously, papers that reported on different analyses from the same study, studies that were follow up analyses of the same population). In cases of related papers, we used a specific set of rules to decide which effect estimate to report and use in the meta-analyses; this rule is described in Section 2.5. 

### 2.4. Chemical Group Classification

We reported results for all chemical groups for which there was information from the available literature. We did not consider exposures to chlorophenols in this paper, since much of the exposure to this chemical group comes from non-agricultural settings. We classified pesticide active ingredients into chemical groups based on Alan Wood’s classification system [13]. 

### 2.5. Reporting of Results for the Systematic Review

From every relevant paper, we extracted an effect estimate for each active ingredient and/or chemical group. We extracted results for associations with NHL, and when available, for associations with subtypes of NHL. 

We used the following algorithm to determine which effect estimates to use:

(1) For related papers that examined the same exposure/outcome association, we used the results from the most complete and updated analysis with the greatest number of participants;
(2) If more than one exposure definition was considered and reported, we used the definition that best represented agricultural exposures (e.g., we selected results for farmers who worked with phenoxy herbicides, instead of results for herbicide applicators, gardeners, or landscapers);
(3) The various papers used different confounder adjustment sets, which were selected based on different criteria. In an effort to use the most unbiased estimate, we extracted the most adjusted effect estimate;
(4) Most papers defined exposure dichotomously. Papers that reported results according to more than two categories used a variety of definitions for the exposure metrics, including duration of use, days/year of use, time since first exposure, and cumulative days of exposure. Because the definitions and metrics used to define categories varied, it was not possible to combine estimates based on multiple categories of exposure in formal meta-analyses. Therefore, for the meta-analyses, we used the result for the dichotomously defined exposure with the greatest number of exposed cases. To assess dose-response relationships, we qualitatively examined results in association with multiple categories;
(5) Some papers only reported results in association with multiple categories of exposure. We extracted these results for the systematic review, since they can be used to qualitatively evaluate trends in association of NHL with active ingredient or chemical group and are important for identifying dose-response relationships;
(6) Some studies only reported estimates of association between pesticide exposures and subtypes of NHL. We abstracted these estimates for presentation and analysis of association of pesticide exposures with NHL subtypes.

We present results from the systematic review sorted by chemical group and, within chemical group, by active ingredient. 

### 2.6. Meta Analysis

#### 2.6.1. Grouping

When possible, we conducted separate meta-analyses for each chemical group and active ingredient. We conducted meta-analyses for associations of these pesticides with NHL and NHL subtypes. Although we abstracted results according to dichotomous exposure and multiple levels of exposure, we conducted formal meta-analyses for dichotomously categorized exposures only.

#### 2.6.2. Analytic Methods

Because we identified a variety of sources of heterogeneity between papers, we decided a priori to calculate meta- risk ratio (RR) estimates and 95% confidence intervals (CIs) using random effect models, allowing between study heterogeneity to contribute to the variance [14,15]. We report I^2^ values, which represent the percentage of the total variance explained by study heterogeneity and measure inconsistency in results. Larger I^2^ values indicate greater inconsistency [15]. We did not perform formal heterogeneity tests; Cochran’s Q statistic has been shown to have low power to detect true heterogeneity across studies, especially in meta-analyses that include a small number of papers [15]. Following recommendations for meta-analyses of observational studies, we also identified possible sources of heterogeneity and used sensitivity analyses to evaluate these, as described in Section 2.6.3 [16]. We evaluate the meta- estimates of association based on the magnitude of the point estimate and interpret the associated 95% CIs as indicators of precision. To aid this interpretation, we have calculated and reported confidence limit ratios (CLRs), which are the ratio of the upper to the lower CI limit [17]. We also present forest plots for meta-analyses to which five or more papers contributed. 

#### 2.6.3. Sensitivity Analysis

We conducted sensitivity analyses to evaluate robustness of our results to the following sources of heterogeneity: study design (case-control versus cohort), gender (male only versus both genders), geographic area, decade of cancer diagnosis, and source of the controls in case-control studies (population-based versus hospital). 

One paper presented results of analyses of women only [18]. Thus, we were not able to conduct a sensitivity analysis for analyses of women; we were able to conduct sensitivity analyses using papers that reported results for men and for men and women. Only two papers reported estimates of association from studies in which controls were drawn from hospitals, and these two studies reported associations of NHL with different pesticides. Therefore, our sensitivity analysis of the control source in case-control studies was restricted to controls drawn from the population. Data from only one cohort study contributed to our meta-analyses. Therefore, we could not restrict meta-analyses to cohort studies only. 

The geographic areas that we investigated separately in sensitivity analyses were North America, the United States, Europe, and Sweden. We selected these because there was more than one study within each area that investigated associations of NHL with a particular pesticide. In addition to maintaining Sweden and the United States in sensitivity analyses of Europe and North America, respectively, we analyzed results from Sweden separately from the rest of Europe, and results from the United States separately from Canada. We conducted these separate analyses because more than one paper reported effect estimates of association with a pesticide from each of these countries, and because we believed effects might be different when separated from the rest of the continent. Although we identified papers from Australia and New Zealand we were not able to analyze these separately because there was not more than one effect estimate of association with an individual pesticide from either country.

We investigated the following diagnosis periods: 1975–1989, 1990–1999, and year 2000 and later. If any part of the diagnosis period overlapped these periods, we included the estimate from the paper in the sensitivity analysis. We selected these periods based on the periods that appeared in the papers that we reviewed and on the different editions of the ICD coding systems [1]. 

After performing meta-analyses for each active ingredient or chemical group, we repeated analyses, removing studies that differed from the others based on the above-described characteristics. In cases when results from individual studies were also represented in papers that analyzed these data pooled with data from other studies, we performed sensitivity analyses by replacing the results from the pooled analyses with the individual studies, or the individual studies with the results from the pooled analyses. 

## 3. Results

### 3.1. Systematic Review

**Figure 1:**
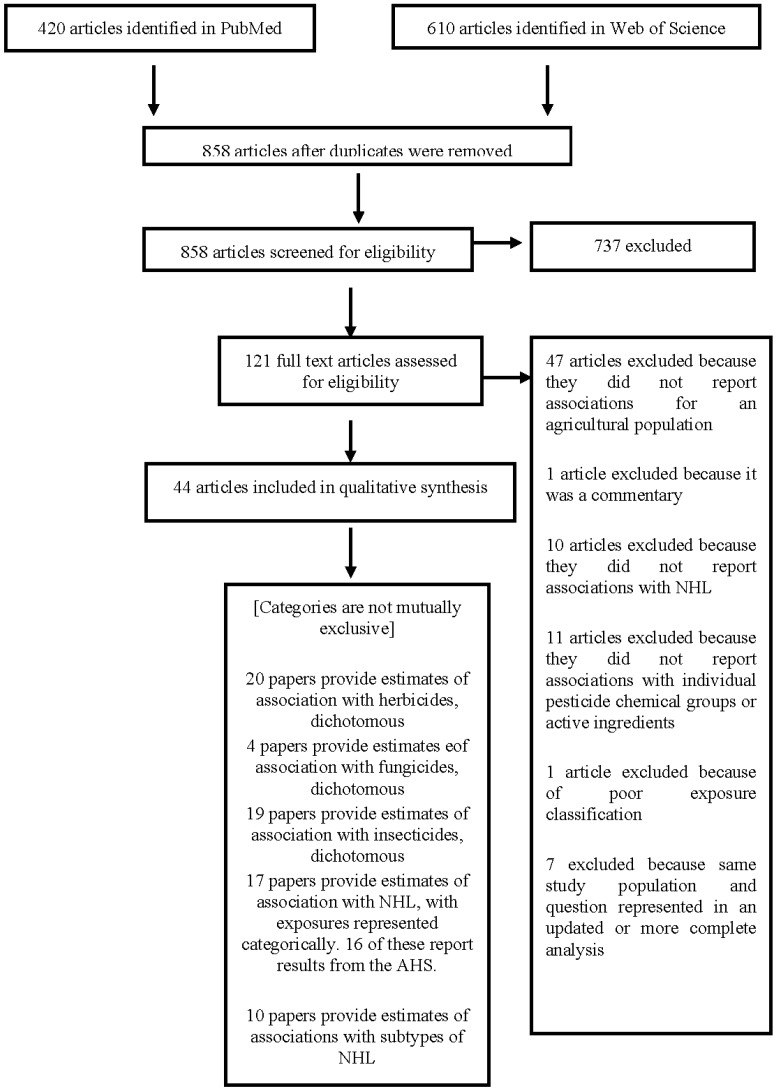
Flow chart showing the articles that were included and excluded in the systematic review, with reasons for the exclusions.

The PubMed and Web of Science searches yielded 858 unique articles (Figure 1). After screening the abstracts and titles, we excluded 737 articles. Of the remaining 121 articles, 47 were excluded because they reported results within a non-agricultural population. We decided to exclude non-agricultural populations because the nature of exposure they receive is different compared to agricultural groups. Because of contamination and production of multiple chemicals simultaneously, it is difficult to determine the exact chemical to which manufacturing cohort participants have been exposed. 

After excluding 27 additional articles because they did not meet one or more of the inclusion criteria described in the methods section, we included 44 papers in our qualitative synthesis. Of these, 20 papers provided estimates of association with herbicide chemical groups or active ingredients, four papers provided estimates of association with fungicides, and 17 with insecticides. 

### 3.2. Summary of Studies from Which Estimates were Extracted

A summary of the 44 papers from which effect estimates were abstracted is presented in Table 1.

**Table 1:** Summary of papers from which effect estimates were extracted.

Author, year, location	Design	Source for controls	Matching	Diagnosis or follow-up period (cancer)	No. Participants	Exposure assessment	Referent category for exposure, exposure definition(s)/metric	Men only	Adjustment set	Pesticides	Reported results by subtype
Barry 2012 [19] Iowa and North Carolina, USA	C (AHS)	NA	NA	1993–2007	53,588	Self-administered questionnaire completed during enrollment and interviewer administered follow-up questionnaire	Referent: No exposure Intensity weighted lifetime exposure days, 15 year lag Intensity weighted lifetime exposure days, no lag	No	Age, gender, race, state of residence, applicator type, enrollment year, cigarette smoking, alcohol consumption, education, family history of cancer, 5 most correlated pesticides	Methyl bromide	No
Baris 1998 [20] Iowa, Kansas, Minnesota, Nebraska, USA	Pooled analysis of 3 CC studies	Population	Matched by race, gender, age, and vital status at the time of interview, year of death for controls matched to deceased cases	Dx period **^1^**: 1979–1983	993 cases/2,918 controls	Telephone interviews (Kansas and Nebraska, USA), In-person interviews (Iowa and Minnesota)	Referent: Non-farmers Used *vs.* did not use on crops and animals, Used *vs.* did not use on crops, Used *vs* did not use on animals Duration of use, in years (1-4, 5-9, ≥10) Days/year of use (≤5, >5)	Yes	Age, state of residence	DDT	Yes
Beane Freeman 2005 [21] Iowa and North Carolina, USA	C (AHS)	NA	NA	1993–2007	23,106	Two self-administered questionnaires	Referent: No exposure Lifetime exposure days Intensity weighted exposure days	Yes	Age, smoking, education, family history of cancer, state of residence, total days of any pesticide application	Diazinon	No
Beane Freeman 2011 [22] Iowa and North Carolina, USA	C (AHS)	NA	NA	1993–2007	36,357	A self-administered questionnaire	Referent 1: No exposure Referent 2: Lowest quartile of exposure Lifetime days of exposure Intensity weighted lifetime days of exposure	No	Age, state, license type, gender, smoking status, alcohol consumption, education, use of most highly correlated pesticides	Atrazine	Yes
Blair 1998 [23] Iowa, Kansas, Minnesota, Nebraska, USA	CC	Population	Matched by race, gender, age, vital status at the time of interview	Dx period **^1^**: 1979–1983	987 cases/2,895 controls	Telephone interviews (Kansas and Nebraska, USA), In-person interviews (Iowa and Minnesota)	Referent: nonfarmer Farmers who ever used Days/year of use (≤4 days, ≥5 days) First lindane use (≥20 years ago, <20 years ago)	Yes	Age, proxy/direct interview, state of residence	Lindane	Yes
Bonner 2010 [24] Iowa and North Carolina, USA	C (AHS)	NA	NA	1993–2005	44,624	Self-administered questionnaires	Referent 1: Nonexposed Referent 2: Lowest tertile of exposure Intensity weighted lifetime exposure days	No	Age, gender, education, family history of cancer, smoking, alcohol, year of enrollment, state of residence, correlated pesticides	Terbufos	No
Bonner 2005 [25] Iowa and North Carolina, USA	C (AHS)	NA	NA	1993–2001	49,877	Self-administered questionnaire	Referent 1: Nonexposed Referent 2: Lowest tertile of exposure Lifetime exposure days Intensity weighted lifetime exposure days	No	Age, gender, education, family history of cancer, smoking, alcohol, year of enrollment, state of residence, exposure to correlated pesticides	Carbofuran	No
Bonner 2007 [26] Iowa and North Carolina, USA	C (AHS)	NA	NA	1993–2002	19,717	Self-administered questionnaire	Referent 1: Nonexposed Referent 2: Lowest tertile of exposure Lifetime exposure days, Intensity weighted lifetime exposure days	No	Age, gender, smoking, alcohol, education, family history of cancer, year of enrollment, state of residence	Malathion	No
Cantor 1992 [27] Iowa and Minnesota, USA	CC	Population	Matched by 5-year age group, vital status at time of interview, and state of residence	Dx period **^1^**: 1979–1983	622 cases/1,245 controls	In-person interviews	Referent: Non-farmers Ever handled, Handled prior to 1965 Handled without protective equipment	Yes	Age, vital status, state, cigarette smoking, family history of lymphohematapoeitic cancer, high risk occupations, high risk exposures	2,4-D, 2,4,5-T, alachlor, atrazine, aldrin, bentazon, butylate, carbofuran, carbaryl, chlordane, chloramben, copper acetoarsenate, cyanazine, coumaphos, diazinon, dicamba, dichlorvos, DDT, famphur, Flyspray, fonofos, glyphosate, heptachlor, lindane, malathion, methoxychlor, metribuzen, nicotine, phorate, popachlor, rotenone, toxaphene, trifluralin, turbufos,	No
Cocco 2013 [28] Multicentre; Czech Republic, France, Germany, Italy, Ireland, Spain	CC	Population (German and Italian centers), Hospital (Czech Republic, French, Irish, Spanish centers)	Matched by gender, 5-year age group, and residence area	1998–2004	2,348 cases/2,462 controls	Structured in-person interviews conducted by trained interviewers, jobs were coded by industrial hygienists; industrial hygienists and occupational experts reviewed the questionnaires and job modules to assess exposures to pesticides (with the help of a crop exposure matrix)	Referent: Never exposed Ever exposed, by level of industrial hygienists’s degree of confidence that the participant was truly exposed to the agent: Any level of confidence High confidence	No	Age, gender, education, study center	Carbamates, OPs, OC, Triazines and triazoles, phenoxy acids, chlorophenols, mancozeb, methomyl, dimethoate, glyphosate, DDT, endosulfan, 2,4-D, MCPA	Only reported for subtypes
Delancey 2009 [29] Iowa and North Carolina, USA	C (AHS)	NA	NA	1993–2004	23,072	Two self-administered questionnaires	Referent: Lowest tertile of exposure Lifetime exposure days Intensity weighted lifetime exposure days	Yes	Age, smoking, alcohol consumption, education, family history of cancer, state of residence, exposure to all pesticides	Metribuzin	No
de Roos 2003 [30] Iowa, Kansas, Minnesota, Nebraska, USA	CC	Population	Matched by race, gender, age, vital status at the time of interview	Dx period **^1^:** 1979–1983	650 cases/1,933 controls	Telephone interviews (Kansas and Nebraska, USA), In-person interviews (Iowa and Minnesota)	Referent: Not exposed Exposed	Yes	Age, study site, use of all other pesticides	Aldrin, bufencarb, carbaryl, carbofuran, chlordane, copper acetoarsenite, coumaphos, DDT, diazinon, dichlorvos, dieldrin, dimethoate, ethoprop, famphur, fly/tick/lice spray, fonofos, heptachlor, lead arsenate, lindane, malathion, methoxychlor, nicotine, phorate, pyrethrins, rotenone, tetrachlorvinphos, toxaphene, terbufos, alachlor, atrazine, bentazon, butylate, chloramben, cyanazine, 2,4-D, dicamba, EPTC, glyphosate, linuron, MCPA, metolachlor, metribuzin, paraquat, propachlor, sodium chlorate, 2,4,5-T, trifluralin	No
de Roos 2005 [31] Iowa and North Carolina, USA	C (AHS)	NA	NA	1993–2001	54,315	Self-administered questionnaire	Referent 1: Never used Referent 2: Lowest tertile of exposure Ever used Cumulative exposure days Intensity weighted exposure days	No	Age at enrollment, education, cigarette smoking, alcohol consumption, family history of cancer, state of residence, other pesticides	Glyphosate	No
Eriksson 2008 [32] Sweden	CC	Population	Matched in 10 year age and gender groups to mirror the age and gender distribution of the cases	Dx period: 1999–2002	1,163 cases/1,016 controls	Telephone interview on life style factors and diseases; Self-administered questionnaire on work history and chemical exposures; follow up telephone interviews to collect incomplete data	Referent: Never exposed Ever exposed, Days of exposure (categorized at the median of the exposure distribution),	No	Age, gender, year of Dx/enrollment	Phenoxyacetic acids, MCPA, 2,4,5-T and/or 2,4-D, glyphosate	Yes
Hardell 2002 [33] Sweden	CC, pooled analysis of two studies, one of hairy cell lymphoma and one of NHL	Population	Matched by age and county	Dx period: 1987–1990 (NHL); 1987–1992 (hair cell lymphoma)	515 cases/1,141 controls	Self-administered questionnai-re supplement-ed by telephone interviews by a trained interviewer when information was unclear	Referent 1: Not exposed Ever exposure, High exposure (>median number of days for exposed participants) Low exposure (<median number of days for exposed participants) Years between first exposure and diagnosis: Referent 2: 1-10years, >10-20 years, >20-30 years, >30 years Years between last exposure and diagnosis: Referent 3: 1-10 years, >10-20 years, >20-30 years, >30 years Decade of exposure	Yes	Study, study area, vital status, age	Phenoxy acids, MCPA, 2,4-D + 2,4,5-T, glyphosate, DDT, mercurial seed dressing, pyrethrins, arsenic	No
Hoar 1986 [34] Kansas, USA	CC	Population	Matched by age and vital status	Dx period: 1976–1982	170 cases of NHL/948 controls (no. included in NHL analysis unclear)	Telephone interviews, with questions on years living/ working on a farm, and herbicides/insecticides used.	Referent: Non-farmers Ever use, Duration of use (years), Frequency of use (days/year), First year of use	Yes	Age	Phenoxyacetic acids, Triazine herbicides, Amide herbicides, Benzoic herbicides, Carbamate herbicides, Trifluralin herbicides, Uracil herbicides	No
Kang 2008 [35] Iowa and North Carolina, USA	C (AHS)	NA	NA	1993–2002	50,127	Self-administered questionnai-res completed during enrollment and interviewer administered follow-up questionnaires	Referent 1: Nonexposed Referent 2: Lowest tertile of exposure Lifetime exposure days, Intensity weighted lifetime exposure days	No	Age at enrollment, education, cigarette smoking, alcohol consumption, family history of cancer, state of residence, top five most highly correlated pesticides	Trifluralin	No
Koutros 2009 [36] Iowa and North Carolina, USA	C (AHS)	NA	NA	1993–2004	49,398	Self-administered questionnaire	Referent: Nonexposed Intensity weighted lifetime exposure days	No	Age, year of enrollment, race	Imazethapyr	No
Koutros 2008 [37] Iowa and North Carolina, USA	C (AHS)	NA	NA	1993–2004	49,762	Self-administered questionnaire	NA	No	Not applicable, since an adjusted effect estimate for an association with NHL was not reported	Dichlorvos	No
Lee 2004 [38] Iowa and North Carolina, USA	C (AHS)	NA	NA	1993–2001	54,383	Self-administered questionnaire	Referent 1: Nonexposed Lifetime exposure days, Intensity weighted lifetime exposure days	No	Age, gender, alcohol consumption, smoking history, educational level, family history of cancer, year of enrollment, state of residence, use of 4 correlated pesticides	Chlorpyrifos	No
Lee 2004 [39] Iowa and North Carolina, USA	C (AHS)	NA	NA	1993–2000	49,980	Self-administered questionnaire	Referent 1: Nonexposed Exposed, Referent 2: Lowest quartile of exposure Lifetime exposure days, Intensity weighted lifetime exposure days	No	Age, sex, alcohol, smoking, education, family history of cancer, enrollment year, state of residence, 5 correlated pesticides	Alachlor	No
Lynch 2009 [40] Iowa and North Carolina, USA	C (AHS)	NA	NA	1993–2004	19,655	Self-administered questionnaire	Referent 1: Nonexposed Referent 2: Lowest tertile of exposure Lifetime exposure days, Intensity weighted lifetime exposure days	No	Age at enrollment, gender, race, smoking status, education, family history of cancer, atrazine, 5 most correlated pesticides	Butylate	No
Lynch 2006 [41] Iowa and North Carolina, USA	C (AHS)	NA	NA	1993–2002	50,800	Self-administered questionnaire	Referent: Lowest tertile of exposure^1^ Lifetime exposure days, Intensity weighted lifetime exposure days	No	Age, race, gender, alcohol consumption, smoking status, education level, family history of cancer, state of residence, 5 most correlated pesticides	Cyanazine	No
Mahajan 2007 [42] Iowa and North Carolina, USA	C (AHS)	NA	NA	1993–2003	21,416	Self-administered questionnai-re	Referent 1: Nonexposed Referent 2: Lowest tertile of exposure Lifetime exposure days, Intensity weighted lifetime exposure days	No	Age, smoking, gender, state of residence, use of 4 correlated pesticides	Carbaryl	No
McDuffie 2001 [43] Six Canadian provinces	CC	Population	Frequency matched by age and province of residence	Dx period: 1991–1994	517 cases/1,506 controls	Self-administered postal questionnai-re followed by telephone interview with participants who had 10 or more hours of pesticide use in lifetime plus a 15% random sample of those with fewer than 10 hours pesticide use	Referent: Not exposed Exposed, Frequency of exposure (days/year)	Yes	Age, providence of residence	2,4-D, mecoprop, MCPA, DiclofopmethylGlyphosate, phosphonic acids, phenoxy herbicides, thiocarbamates, diallate, dicamba, dinitroaniline, trifluralin, carbaryl, carbofuran, methomyl, carbamate insecticides, organochlorine insecticides, chlordane, lindane, aldrin, methoxychlor, DDT, Captan, vitavax, aldehyde, formaldehyde, mercury dust, mercury liquid, malathion, carbon tetrachloride	
Miligi 2006 [44] Italy	CC	Population	Stratified by gender and 5-year age groups	Dx period: 1991–1993	1,145 cases/1,232 controls	In-person interviews, including questions on crops grown and whether pesticides were used combined with exposure matrix	Reference: Those who never worked in agriculture Overall exposure, Probability of use >low and lack of protective equipment	No	Age, gender, area	Phenoxy herbicides,2,4-D, MCPA	No
Mills 2005 [45] California	CC	Same source as the cases (United Farm Workers of America cohort)	Matched by gender, hispanic ethnicity and +/− one year of birth	Dx period: 1988–2001	60 cases/300 controls	Work histories combined with exposure matrix	Reference: Low use High use	No	Age, gender, length of union affiliation, date of first union affiliation	Methyl bromide, diazinon, malathion, dichloro-propane, captan, simazine, chlrothalonil, mancozeb, methyl parathion, nitrofen, propyzamide, toxaphene, trifluralin, 2,4-D, maneb	No
Orsi 2009 [46] France	CC	Hospital	Matched by center, age +/− 3 year, gender	2000–2004	244 cases/436 controls	Self-administered questionnai-res, followed by face to face interviews with trained staff, and review of interviews by experts to verify logical consistency with pesticide product availability, geographic location, *etc.*	Reference: Nonexposed Exposed	No	Age, center, socioeconomic characteristic (white collar vs blue collar)	Organochlorine insecticides, organophosphorus insecticides, pyrethrin, carbamate fungicides, imide fungicides, triazole fungicides, phenoline herbicides, phenoxy herbicides, picoline herbicides, triazine herbicides, amide herbicides, urea herbicides, quaternary ammonium herbicides, glyphosate	Yes
Pahwa M 2012 [47] Six Canadian provinces	CC	Population	Frequency matched by age and province of residence	Dx period: 1991–1994	513 cases/ 506 controls	Self-administered postal questionnaire followed by telephone interview with participants who had 10 or more hours of pesticide use in lifetime plus a 15% random sample of those with fewer than 10 hours pesticide use	Reference: No use Use	Yes	Age, province of residence, respondent type (self or proxy), diesel oil exposure	OC insecticides, DDT, OP insecticides, malathion, phenoxy herbicides, MCPA, mecoprop,2,4-D	No
Pearce 1987 [48] New Zealand	CC	Cancer registry	Matched by year of cancer registration and age (± 2 years)	Dx period: 1977–1981	183 cases/338 controls	Telephone interviews	Reference: Nonexposed Used any agricultural chemical spray in a farming setting	Yes	Decade of birth, type of interview respondent (self or relative)	Phenoxy herbicides	No
Persson 1989 [49] Sweden	CC	Population	Unmatched	Dx period: 1964–1986	106 cases/275 controls	Self-administered questionnaire	Reference: Not exposed Exposed	No	Age, date of Dx, gender, farming, exposure to fresh wood, other exposures associated with at least a doubled risk for hodgkins disease or NHL	Phenoxy herbicides, DDT	No
Persson 1993 [50] Sweden	CC	Population	Unmatched	Dx period: 1975–1984	93 cases/204 controls	Self-administered questionnaires	Reference: Not exposed Exposed	No	Age, other exposures investigated with OR ≥2 or significantly below unity and with at least 5 exposed subjects	Phenoxy herbicides, DDT	No
Purdue 2007 [51] Iowa and North Carolina, USA	C (AHS)	NA	NA	1993–2002	51,011	Self-administered questionnaire	Reference 1: Never use/unexposed Ever use Lifetime days of exposure Intensity weighted lifetime days of exposure	No	Age, sate, gender, education level, smoking status, alcohol use, family history of cancer, lifetime days of total pesticide application	OC insecticides, aldrin, chlordane, DDT, dieldrin, heptachlor, lindane, toxaphene	No
Rafnsson 2006 [52] Iceland	CC	Non-cases from cohort of sheep owners	Unmatched	Dx period: 1966–2003	45 cases/221 controls	Records of sheep owned, used as a proxy measure for dermal exposure from sheep dipping; sheep dipping used as a proxy for exposure to hexa-chlorocyclohexane, which is a mixture of different isomers containing around 15% lindane. <100 sheep owned was used to indicate unexposed	Referent: <100 sheep ≥100 sheep , Categories of number of sheep owned: 100-199 sheep, 200-683 sheep	Yes	Age	Hexachlorocyc-lohexane	No
Rusiecki 2009 [53] Iowa and North Carolina, USA	C (AHS)	NA	NA	1993–2004	49,093	Self-administered questionnaire	Referent: Nonexposed Lifetime days of exposure Intensity weighted	No	Age, gender, race, family history of cancer, cigarette smoking, state of residence, enrollment year	Permethrin	No
Rusiecki 2006 [54] Iowa and North Carolina, USA	C (AHS)	NA	NA	1993–2002	50,193	Self-administered questionnaire	Referent: Lowest tertile of exposure Lifetime days of exposure Intensity weighted lifetime days of exposure	No	Age, gender, race, smoking, alcohol, applicator status, family history of cancer, sate of residence, most highly correlated pesticides	Metolachlor	No
van Bemmel 2008 [55] Iowa and North Carolina, USA	C (AHS)	NA	NA	1993–2004	48,378	Self-administered questionnaire	Referent: No exposure Lifetime days of exposure Intensity weighted lifetime days of exposure	Yes	Age, race, smoking, alcohol use, applicator type, family history of cancer, state of residence, total days of pesticide use	EPTC	No
Waddell 2001 [56] Iowa, Kansas, Minnesota, Nebraska, USA	Pooled analysis of 3 CC studies	Population	Matched by race, gender, age, and vital status at the time of interview, year of death for controls matched to deceased cases	Dx period ^1^: 1979–1983	748 cases/2,236 controls	Telephone interviews (Kansas and Nebraska, USA), In-person interviews (Iowa and Minnesota)	Referent: Non-farmers Ever Used First used Years used Days/year of use Protective gear	Yes	Age, state of residence, respondent type (proxy or direct)	OP insecticides, dichlorvos, trichlorfon, dimethoate, diazinon, disulfoton, ethoprop, malathion, phorate, terbufos, chloropyrifos, coumaphos, crufomate, runnel, tetrachlorvinphos, fensulfothion, famphur, fonofos, parathion	Yes
Woods 1987 [57] Washington state, USA	CC	Population	Matched by vital status and 5-year age group	Dx period: 1981–1984	746 cases/910 controls	In-person interviews about occupational history and self-reported chemical exposure	Referent: No exposure Farming exposures to phenoxy herbicides Any exposure to DDT Any exposure to chlordane Estimated intensity of occupational exposure to phenoxy herbicides: Low/medium/high	Yes	Age	Phenoxy herbicides, DDT, Chlordane	No
Zahm 1990 [58] Nebraska, USA	CC	Population	Matched by race, gender, vital status, age	Dx period: 1983–1986	201 cases/725 controls	In-person interviews about agricultural pesticide use	Referent: Never lived or worked on a farm Mixed or applied Days/year mixing or applying Years used on a farm First year of use	Yes	Age	2,4-D	No
Zahm 1993 [59] Iowa, Kansas, Minnesota, Nebraska, USA	Pooled analysis of 3 CC studies	Population	Matched by race, gender, age, vital status at the time of interview	Dx period **^1^**: 1979–1983	993 cases/2,918 controls	Telephone interviews (Kansas and Nebraska, USA), In-person interviews (Iowa and Minnesota)	Referent: Non-farmers Used atrazine **^1^** Personally handled Used but did not handle Duration of use (years) Days/year handled Year of first use	Yes	Age, state	Atrazine	Yes
Zahm 1993 [18] Nebraska, USA	CC	Population	Matched by race, gender, vital status, and age (5 year age groups)	Dx period: 1983–1986	119 cases/471 controls	In-person interviews about agricultural pesticide use	Referent: women who never lived or worked on a farm Used on farm	No (women only)	Age	Phenoxy herbicides, triazine herbicides, amide herbicides, benzoic acid herbicides, carbamate herbicides, trifluralin herbicides, chlorinated hydrocarbons, carbamate insecticides, OP insecticides	No
Zheng 2001 [60] Nebraska, USA, Iowa and Minnesota, Kansas	Pooled analysis of 3 CC studies	Population	Matched by gender, age, race, vital status, state of residence	Dx period **^1^**: 1979–1983	985 cases/2,895 controls	In-person interviews about agricultural pesticide use	Referent: Non-farmers Used Personally handled Year since first use Years of use Days/year of use	Yes	Age, type of respondent (proxy or direct), state of residence, first-degree family history of cancer, use of hair dye, use of private wells, tobacco smoking	Carbaryl, carbamate herbicides, carbamate insecticides	Yes

Notes: 2,4-D, 2,4-Dichlorophenoxyacetic acid; 2,4,5-T, 2,4,5-Trichlorophenoxyacetic acid; AHS, Agricultural Health Study; C, cohort study; CC, case-control study; DDT, dichlorodiphenyltrichloroethane; DX, Diagnosis; EPTC, s-ethyl dipropylthiocarbamate; MCPA, 2-methyl-4-chlorophenoxyacetic acid; OC, organochlorine; OP, organophosphorus; **^1^** Diagnosis period varied by state: July 1983–June 1986 (Nebraska, USA), October 1980–September 1982 (Minnesota), March 1981-October 1983 (Iowa), 1979–1981 (Kansas).

#### 3.2.1. Studies Conducted in the United States

Nineteen papers [19,21,22,24,25,26,29,31,35,36,38,39,40,41,42,51,53,54,55] report results from analyses of data from the Agricultural Health Study, which is a prospective cohort study of licensed pesticide applicators and their spouses living in Iowa and North Carolina, USA. Enrollment began in 1993 and the study is still ongoing [61]. The number of participants included in the analyses varied due to exclusions and completeness of exposure data. The last year of follow-up was defined by the last date on which the incident cancers were identified. At enrollment, participants completed a questionnaire in which they provided historical data on exposure to pesticides. They were also given a take home questionnaire to complete. Most analyses of the Agricultural Health Study data classified active ingredient exposures using two metrics: (1) lifetime exposure days, defined as number of years of use x number of days used per year; and (2) intensity-weighted lifetime exposure days, which was defined as years of use x number of days used per year x personal protective equipment use x intensity level, which incorporates factors that modify pesticide exposure, such as mixing status, application method, equipment repair status. Four papers [31,38,39,51] also reported associations using ever/never use categories; we used these estimates in the meta-analyses. 

Six papers reported results from pooled analyses of three case-control studies that were conducted by the USA National Cancer Institute [20,23,30,56,59,60], in Iowa and Minnesota, Kansas, and Nebraska. Diagnosis periods for NHL ranged from 1979 to 1986, depending upon the study. In all studies, population based controls were frequency matched to cases by race, sex, age, and vital status at the time of the interview, and lifetime exposure to pesticides was assessed via telephone interviews. Using these pooled data, De Roos *et al.* [30] examined associations of NHL with 47 active ingredients. The authors investigated pesticides for which there was exposure data from all three studies and to which at least 20 participants were exposed. They used standard logistic regression to model the association of NHL with the multiple pesticides, simultaneously. These analyses were restricted to participants with complete information on all of the pesticides. Other papers reported results from analyses of these pooled data. Baris *et al.* [20] examined associations with dichlorodiphenyltrichloroethane (DDT), Blair *et al.* [23] with lindane, Zahm *et al.* [59] with atrazine, Waddell *et al.* [56] with organophosphates, and Zheng *et al.* [60] with carbamates. We also extracted results from analyses of the individual studies. Using data from the study in Iowa and Minnesota, Cantor *et al.* [27] examined associations with multiple pesticides. In Kansas, Hoar *et al.* [34] examined associations with exposures to various herbicides. In Nebraska, Zahm *et al.* [58] examined associations with 2,4-Dichlorophenoxyacetic acid (2,4-D). 

In a population based case-control study in western Washington State, USA Woods *et al*. [57], examined associations between phenoxy exposure and NHL. Controls were group matched to cases diagnosed 1981–1984, based on vital status and age. Lifetime occupational histories and self-reported pesticide chemical exposures were ascertained using in-person interviews. The authors reported exposure to phenoxy herbicides by occupational type. We extracted the result for farming exposure to phenoxy herbicides. Exposures to DDT and chlordane were reported as ever/never, but they were not stratified by occupation. 

We also extracted results from a USA based case-control study nested in a cohort of primarily Hispanic members of the California farm worker labor union [45]. Cases were diagnosed 1988–2001. Controls were selected from the same cohort as cases and matched on the basis of gender, Hispanic ethnicity, and year of birth. Pesticide exposure was defined as low *versus* high use, with the category cut-points based on the distribution of use of the top 15 pesticides. To estimate exposure, union job history data that described crops farmed in a given month/year and county was combined with data collected by the California Pesticide Databank that describes pesticides used on a crop in a given county and time period. 

#### 3.2.2. Canadian Studies

Two papers reported results from the Cross-Canada Study of Pesticides and Health, which was a case control study conducted in six Canadian provinces [43,47]. Population based controls were frequency matched to NHL cases, diagnosed 1991–1994, based on age and province of residence. Detailed information on specific pesticide use was ascertained by telephone interviews. The questionnaires used for this study were based on the one used in the USA National Cancer Institute led case-control studies [20,23,30,56,59,60] in Nebraska [18,58] and Kansas [34]. McDuffie *et al.* [43] and Pahwa *et al.* [47] present results from some of the same analyses with the same population. When the same analysis was reported in both papers we selected the effect estimate from the paper by Pahwa *et al.* [47] because the authors excluded four NHL cases based on pathology review that occurred subsequent to the analyses reported in McDuffie *et al.* [43]. 

#### 3.2.3. European Studies

Four papers [32,33,49,50] reported results from distinct case-control studies conducted in Sweden. The papers by Eriksson *et al.* [32] and Hardell *et al.* [33] reported analyses from population based case-control studies; case diagnosis periods were 1999–2002 and 1987–1992, respectively. A complete lifetime occupational and chemical exposure history was ascertained using self-administered questionnaires followed by telephone interviews when clarification was needed. The two studies by Persson *et al.* [49,50] report results from unmatched population based case-control studies; the results reported from the paper published in 1993 [50] were performed in an adjacent region of Sweden to the area represented in the earlier paper [49]. They examined the association of NHL with various occupational exposures, including phenoxy herbicides and DDT. Case diagnosis periods were 1964–1986 and 1975–1984, respectively. 

We extracted results from papers that report results from analyses of data collected in France [46], Italy [44], Iceland [52], and multiple European centers that form parts of the EPILYMPH study [28]. All of these studies were case-control in design. In France [46], cases (diagnosed 2000–2004) and controls were recruited in the same hospitals. Exposure was assessed using self-administered questionnaires, followed by face-to-face interviews in which participants reported information about farms on which they worked for a minimum of six months; they reported information about location, period, crops and animals farmed, name of pesticides mixed or sprayed, duration and number of pesticide applications. Pesticide exposure was classified as possible or definite; the referent category included people never exposed to the pesticide. In the Italian study [44], cases were diagnosed from 1991 to 1993. Participants were interviewed about agricultural work, crop diseases, pesticides used to treat diseases, frequency of pesticide treatments, period of treatment, protective equipment used, means of application, and re-entry tasks. Exposure was classified into low, medium, and high probabilities of use. The Icelandic case-control study [52] was nested in a cohort of male sheep owners. The authors included cases diagnosed 1966–2003. Paper records on sheep dipping in hexachlorocyclohexane, an organochlorine insecticide that contains lindane, were used as a proxy measure for exposure; records were available for the period 1962–1980. Number of sheep owned was used as a surrogate measure for exposure. In the EPILYMPH study [28], in-person interviews were conducted to ascertain detailed job histories, including information about farm size, crops farmed, pests treated, types and frequency of pesticides used, protective equipment, and re-entry tasks. Industrial hygienists classified pesticide exposure as possible, probable, or certain. In analysis, contrasts were made between high confidence of ever lifetime exposure *versus* never exposure, and any level of confidence of ever exposure *versus* never exposure. 

#### 3.2.4. Studies from Australia and New Zealand

Only two papers reported results from analyses of studies conducted outside of North America and Europe. Pearce *et al.* [48] reported analyses of data from a New Zealand based case-control study of agricultural exposures. Cases were diagnosed 1977–1981. Telephone interviews were used to ascertain lifetime occupational history and work with chemicals (phenoxy herbicides). In analysis, Pearce *et al.* [48] stratified phenoxy herbicide exposure by occupation (farming, forestry, railway work, *etc.*). We extracted the estimate of association with any phenoxy herbicide exposure in farming occupations. In Australia [62], Fritschi *et al.* enrolled incident NHL cases diagnosed between 2000 and 2001. They matched controls to cases based on age, gender, and region of residence. In structured telephone interviews, participants provided occupational histories. Occupational hygienists reviewed the responses to these questions and, with the help of a pesticide crop matrix, assigned likelihood of exposure to pesticides (probable, possible, no exposure), level of exposure, and frequency of exposure. These assignments were combined to classify participants’ lifetime amount of exposure (substantial, meaning the person was probably exposed to the substance at a medium or high level for more than five 8-h days per year for a combined total of five years, nonsubstantial, or none).

#### 3.2.5. Gender

Nineteen of the papers reported results from analyses that were restricted to men only [20,21,23,27,28,29,30,33,34,43,47,48,52,55,56,57,58,59,60]. One paper reported results from an analysis that was restricted to women [18]. The other papers reported results from analyses of study populations with men and women; in the analytic models reported in these papers, gender was treated as a confounder. 

#### 3.2.6. Covariates

In all papers, age was included in models to adjust for potential confounding. Location (state of residence, study center) was also a common adjustment factor. Other variables that were included in models as covariates were race, smoking status, alcohol consumption, correlated pesticides, education level, year of study enrollment, family history of cancer (all cancers or lympho-hematopoetic), other environmental risk factors for NHL (e.g., gasoline exposure), and type of respondent to the interview used for exposure assessment (direct or proxy). 

#### 3.2.7. Reference Groups

In the majority of papers reviewed, the reference group contained farmers and non-farmers who were not exposed to the pesticide. However, there were exceptions to this, either because of study design or analytic decisions.

By design, all participants in the Agricultural Health Study were either pesticide applicators or spouses of applicators. Most of the analyses from this cohort contrasted exposed participants with two different referent groups: (1) participants with no exposure to the pesticide; and (2) participants in the lowest category of exposure. Similarly, all of the participants in the California based study reported in Mills *et al.* [45] were farm workers. The referent group in this analysis consisted of those with estimated low use of the pesticide being analyzed. Both cases and controls in the Icelandic study on which Rafnsson *et al.* [52] reported were sheep owners; people who owned <100 sheep made up the reference group. 

By contrast, in some papers, the authors defined the reference group as those who neither worked nor lived on a farm. Miligi *et al.* [44] defined the referent group as participants who never worked in agriculture. Similarly, in papers reporting analyses of the case-control studies in Iowa, Minnesota, Nebraska, and Kansas, the referent group was defined as participants who never worked or lived on a farm. The exception to this was the paper by De Roos *et al.* [30]; the authors used pooled data from these case-control studies but defined the referent group as farmers and non-farmers who never used the pesticide being considered. 

#### 3.2.8. Exposure Period and Definition

Pesticide exposure periods and definitions varied, also. For the most part, papers investigated associations of NHL with ever lifetime pesticide exposure. However, some were more specific in their definition, and not all papers used the ever lifetime exposure metric. 

In the cohort of California based union farm workers, Mills *et al.* [45] assessed pesticide exposure in the two to three year decade period prior to cancer diagnosis or enrollment. In Canada, McDuffie *et al.* [43] and Pahwa *et al.* [47] defined pesticide exposure as ever *versus* never lifetime use of pesticides for at least 10 h. In Sweden, Eriksson *et al.* [32] and Hardell *et al.* [33] required participants to have worked with the pesticide for a minimum of eight hours in a day, and the pesticide exposure was required to have occurred at least one year prior to the time of diagnosis or enrollment. Persson *et al.* [49,50] only classified as exposed those participants who were exposed to the chemical for at least one year, five to 45 years prior to case diagnosis. In the Italian study described by Miligi *et al.* [44], an agricultural pesticide questionnaire was only administered to participants who had worked on a farm for at least six months; presumably, therefore, anyone who had worked with pesticides but worked on a farm for less than six months was excluded from the exposed group. In the Icelandic study that Rafnsson *et al.* [52] described, records on sheep ownership, which were used to estimate lindane exposure, were available for the period 1962–1980; however, the cancer diagnosis period was 1966–2003. 

### 3.3. Individual Effect Estimates and Dose Response Relationships

Table 2 presents effect estimates from studies in which chemical exposures were represented using multiple categories. Strong dose response relationships were generally absent; most analyses that examined associations with multiple categories of exposure derived imprecise estimates with wide confidence intervals. McDuffie *et al.* [43] and Eriksson *et al.* [32] observed increased odds of NHL in association with a greater number of days/year of glyphosate exposure. De Roos *et al.* [31] did not observe a similar relationship in analyses of Agricultural Health Study data. McDuffie *et al.* [43] observed elevated effect estimates in association with exposure to 2,4-D; however, they did not observe a dose-response relationship with days/year exposed. In analyses of Agricultural Health Study data, Lynch *et al.* [40] observed a nearly three-fold increase in the rate of NHL among those with ≥26 lifetime- and intensity-weighted exposure days to butylate, although the rate ratio comparing those with one to 25 lifetime exposure days to non-exposed applicators was close to the null.

**Table 2:** Effect estimates from studies that investigated associations between non-Hodgkin lymphoma and herbicides, insecticide, and fungicide exposures classified using multiple categories.

Author, date	Pesticide	Category of exposure	N exposed	Effect estimate, 95% CI
**HERBICIDES**				
Amide herbicides				
Lee 2004 [39]	Alachlor	Lifetime exposure days ^1^		Rate ratio, 95% CI:
		Quartile 1, 0.1–19.9	5	1.0, Referent
		Quartile 2, 20.0–56.0	4	0.6, 0.1–2.5
		Quartile 3, 56.1–116.0	8	1.5, 0.4–5.4
		Quartile 4, ≥116.1	10	1.1, 0.3–4.4
				P for trend: 0.5
		Intensity weighted exposure days ^1^		
		Quartile 1, 0.1–101.9	5	1.0, Referent
		Quartile 2, 102.0–253.1	3	0.6, 0.1–3.4
		Quartile 3, 253.2–710.4	10	2.4, 0.7–8.8
		Quartile 4, ≥710.5	9	1.4, 0.3–6.1
				P for trend: 0.4
Rusiecki 2006 [54]	Metolachlor	Lifetime exposure days ^1^		Rate ratio, 95% CI:
		Tertile 1, ≤20	14	1.0, Referent
		Tertile 2, 21–56	11	0.8, 0.3–1.7
		Tertile 3, >56	11	0.7, 0.3–1.7
				P for trend: 0.5
		Intensity-weighted lifetime exposure days ^1^		
		Tertile 1, ≤20	13	1.0,Referent
		Tertile 2, 21–56	10	0.7, 0.3–1.7
		Tertile 3, >56	13	1.0, 0.4–2.7
				P for trend: 0.7
Dinitroaniline herbicides				
Kang 2008 [35]	Trifluralin	Lifetime days of exposure ^2^		Rate ratio, 95% CI
		Non-exposed	53	1.0, referent
		Tertile1, 1-24.4	17	0.9, 0.5–1.5
		Tertile 2, 24.5-108.4	23	1.0, 0.6–1.8
		Tertile 3, Lower half, 108.5-224.75	6	0.6, 0.2–1.4
		Tertile 3, Upper half, >224.75	4	0.6, 0.2–1.7
				P for trend: 0.2
		Intensity weighted lifetime days ^2^		
		Tertile 1, 0-162.1	15	0.7, 0.4–1.4
		Tertile 2, 162.2-593	20	1.1, 0.8–2.9
		Tertile 3, Lower half, 593.1-1176.0	9	0.9, 0.4–2.0
		Tertile 3, Upper half, >1176.0	4	0.4, 0.1–1.1
				P for trend: 0.1
Glyphosate				
McDuffie 2001 [43] ^3^	Glyphosate	Days/year of exposure		OR, 95% CI:
		Unexposed	466 cases/1,373 controls	1.0, Referent
		>0–≤2	28 cases/97 controls	1.0, 0.6–1.6
		>2	23 cases/36 controls	2.1, 1.2–3.7
De Roos 2005 [31] ^3^	Glyphosate			
		Lifetime days of exposure ^2^		Rate ratio, 95% CI:
		Tertile 1, 1–20	29	1.0, Referent
		Tertile 2, 21–56	15	0.7, 0.4–1.4
		Tertile 3, 57–2678	17	0.9, 0.5–1.6
				P for trend: 0.7
		Intensity weighted exposure days ^2^		
		Tertile 1, 0.1–79.5	24	1.0, Referent
		Tertile 2, 79.6–337.1	15	0.6, 0.3–1.1
		Tertile 3, 337.2–1,824.1	22	0.8, 0.5–1.4
				P for trend: 1.0
Eriksson 2008 [32] ^3^	Glyphosate			OR, 95% CI:
		Days of exposure ^4^		
		>0–≤10 days	12 cases/9 controls	1.7, 0.7–4.1
		>10 days	17 cases/9 controls	2.4, 1.0–5.4
Imidazolinone herbicides				
Koutros 2009 [36]	Imazethapyr	Intensity weighted exposure days ^5^		Rate ratio, 95% CI:
		No exposure:	80	1.0, Referent
		Tertile 1, <54.1	15	1.0, 0.6–1.7
		Tertile 2, 54.1–<152.9	13	0.9, 0.5, 1.6
		Tertile 3, lower half, 152.9–<311.9	7	0.8, 0.3–1.8
		Tertile 3, upper half, ≥311.9	11	1.4, 0.8–2.7
				P for trend: 0.4
Phenoxy herbicides				
Fritschi 2005 [62]	Phenoxy herbicides, group	Degree of pesticide exposure ^6^		OR, 95% CI:
		None	679 cases/677 controls	1.0, Referent
		Nonsubstantial	10 cases/14 controls	0.7, 0.3–1.7
		Substantial	5 cases/3 controls	1.8, 0.4–7.4
Eriksson 2008 [32] ^3^	Phenoxy herbicides, group	Days of exposure ^4^		
		>0–≤45 days	32 cases/13 controls	2.8, 1.5–5.5
		>45 days	15 cases/13 controls	1.3, 0.6–2.7
Hardell 2002 [33] ^3^	Phenoxy herbicides, group			OR, 95% CI:
		Number of days of exposure		
		Not exposed	NR	1.0, Referent
		Low	NR	1.7, 1.0–2.7
		High	NR	1.7, 1.0–2.7
		Years between first exposure and diagnosis		
		1–10	NR	-
		>10–20	NR	2.9, 1.1–7.7
		>20–30	NR	1.5, 0.9–2.8
		>30	NR	1.5, 0.9–2.4
		Years between last exposure and diagnosis		
		1–10	NR	3.2, 1.6–6.7
		>10–20	NR	2.1, 1.0–4.1
		>20–30	NR	1.0, 0.5–1.8
		>30	NR	1.3, 0.6–2.6
		Decade of exposure		
		1940s	4 cases/6 controls	1.5, 0.4–5.2
		1950s	35 cases/53 controls	1.4, 0.9–2.3
		1960s	43 cases/58 controls	1.7, 1.1–2.6
		1970s	32 cases/33 controls	2.4, 1.4–4.0
		1980s	16 cases/33 controls	3.3, 1.5–7.1
Eriksson 2008 [32] ^3^	MCPA	Days exposed ^4^		
		≤32	15 cases/5 controls	3.8, 1.4–10.5
		>32	6 cases/4 controls	1.7, 0.5–6.0
Hardell 2002 [33] ^3^	MCPA			OR, 95% CI:
		Number of days of exposure		
		Not exposed	NR	1.0, Referent
		Low	NR	1.9, 0.8–4.6
		High	NR	3.6, 1.5–9.1
		Years between first exposure and diagnosis		
		1–10	NR	-
		>10–20	NR	5.4, 1.6–21
		>20–30	NR	0.9, 0.2–3.0
		>30	NR	3.8, 1.5–10.0
		Years between last exposure and diagnosis		
		1–10	NR	3.5, 1.6–8.0
		>10–20	NR	2.3, 0.6–9.1
		>20–30	NR	0.9, 0.1–4.4
		>30	NR	-
McDuffie 2001 [43] ^3^	Mecoprop	Days/year exposed		
		Unexposed	464 cases/1,425 controls	1.0, Referent
		>0–≤2	31 cases/48 controls	2.3, 1.4–3.7
		≥2	22 cases/33 controls	2.1, 1.2–3.6
Hardell 2002 [33] ^3^	2,4-D + 2,4,5-T			OR, 95% CI:
		Number of days of exposure		
		Low	NR	1.9, 1.1–3.2
		High	NR	1.2, 0.7–2.1
		Years between first exposure and diagnosis		
		1–10	NR	-
		>10–20	NR	2.9, 0.8–11.0
		>20–30	NR	1.9, 1.0–3.5
		>30	NR	1.2, 0.7–1.9
		Years between last exposure and diagnosis		
		1–10	NR	4.3, 1.1–21.0
		>10–20	NR	1.9, 0.9–3.8
		>20–30	NR	0.9, 0.1–4.4
		>30	NR	1.4, 0.7–2.9
Eriksson 2008 [32] ^3^	2,4,5-T and/or 2,4-D	Days exposed ^4^		OR, 95% CI:
		Non-exposed		1.0, Referent
		≤29	21 cases/11 controls	2.1, 1.0–4.4
		>29	12 cases/10 controls	1.3, 0.6–3.1
Zahm 1990 [58] ^3^	2,4-D			OR, 95% CI:
		Never lived/worked on a farm	54 cases/184 controls	1.0, Referent
		Days/year mixing or applying			
		1–5	16 cases/44 controls	1.2, 0.6–2.4
		6–20	12 cases/25 controls	1.6, 0.7–3.6
		21+	3 cases/4 controls	3.3, 0.5–22.1
		Unknown	12 cases/25 controls	-
				P for trend: 0.1
		Years used on farm		
		1–5	3 cases/12 controls	0.9, 0.2–3.6
		6–15	11 cases/15 controls	2.8, 1.1–7.1
		16–20	3 cases/18 controls	0.6, 0.1–2.1
		21+	13 cases/33 controls	1.3, 0.6–2.7
		Unknown	15 cases/29 controls	-
				P for trend: 0.3
		First year of use		
		Prior to 1945	8 cases/21 controls	1.4, 0.5–3.5
		1946–1955	13 cases/39 controls	1.1, 0.5–2.3
		1956–1965	5 cases/8 controls	2.1, 0.6–7.7
		1965–1986	4 cases/12 controls	1.3, 0.3–4.9
		Unknown year	13 cases/18 controls	-
				P for trend: 0.2
McDuffie 2001 [43] ^3^	2,4-D	Days/yr exposed		OR, 95% CI:
		Unexposed	406 cases/1,213 controls	1.0, Referent
		>0–≤2	55 cases/160 controls	1.2, 0.8–1.6
		>2–≤5	36 cases/82 controls	1.4, 0.9–2.1
		>5–≤7	9 cases/20 controls	1.4, 0.6–3.2
		>7	11 cases/31 controls	1.2, 0.6–2.5
Thiocarbamate herbicides				
Zheng 2001 [60] ^3^	Butylate			OR, 95% CI
		Non-farmers	243 cases/775 controls	1.0, Referent
		Years since first use		
		<20	34 cases/56 controls	1.5, 0.9–2.4
		≥20	4 cases/10 controls	1.1, 0.3–3.7
		Years of use		
		<7	21 cases/35 controls	1.5, 0.9–2.8
		≥7	20 cases/37 controls	1.5, 0.8–2.7
		Days/year of use		
		<5	3 cases/5 controls	2.6, 0.6–11.1
		≥5	2 cases/2 controls	4.7, 0.6–34.5
Lynch 2009 [40]	Butylate	Lifetime exposure days ^5^		Rate ratio, 95% CI:
		No exposure	27	1.0, Referent
		Low exposure, 1–25	6	0.9, 0.4–2.0
		High exposure, ≥26	12	2.9, 1.5–5.8
				P for trend: 0.0
		Intensity weighted exposure days ^5^		
		No exposure	27	1.0, Referent
		Low exposure, 1–157	5	0.8, 0.3–2.0
		High exposure, ≥158	13	2.9, 1.5–5.5
				P for trend: 0.0
Van Bemmel 2008 [55]	EPTC	Lifetime exposure days ^5^		Rate ratio, 95% CI:
		No exposure	83	1.0, Referent
		Tertile 1, 1–9	10	1.2, 0.6–2.3
		Tertile 2, 10–49	7	1.5, 0.7–3.2
		Tertile 3, 50+	5	0.8, 0.3–2.0
				P for trend:0.7
		Intensity-weighted lifetime exposure days ^5^		Rate ratio, 95% CI:
		No exposure	83	1.0, Referent
		Tertile 1, 1–47	8	1.4, 0.7–2.8
		Tertile 2, 48–111	4	0.9, 0.3–2.5
		Tertile 3, 112+	10	1.1, 0.6–2.1
				P for trend:0.9
Zheng 2001 [60] ^3^	EPTC + Protectant			OR, 95% CI:
		Non-farmers:		1.0, Referent
		Years since first use		
		<20	19 cases/34 controls	1.7, 0.9–3.1
		≥20	0 cases/1 control	-
		Years of use		
		<7	15 cases/20 controls	2.2, 1.1–4.4
		≥7	7 cases/26 controls	1.0. 0.4–2.4
		Days/year of use		
		<5	7 cases/12 controls	2.2, 0.8–5.8
		≥5	1 case/5 controls	0.9, 0.1–7.7
Triazine herbicides				
Lynch 2006 [41]	Cyanazine	Lifetime exposure days ^5^		Rate ratio, 95% CI
		Tertile 1, 1–16	9	1.0, Referent
		Tertile 2, 17–56	18	1.6, 0.7–3.5
		Tertile 3, ≥57	9	1.3, 0.5–3.4
				P for trend: 1.0
		Intensity-weighted exposure days ^5^		
		Tertile 1, 1–83	10	1.0, Referent
		Tertile 2, 84–314.35	12	1.3, 0.6–3.0
		Tertile 3, ≥315.35	13	1.4, 0.6–3.4
				P for trend: 0.5
Zahm 1993 [59] ^3^	Atrazine			OR (95% CI not presented)
		No use	445 cases/1507 controls	1.0, Referent
		Years of use		
		1–5	4 cases/14 controls	0.4
		6–15	5 cases/20 controls	0.5
		16–20	5 cases/8 controls	0.6
		≥21	7 cases/11 controls	0.8
		Days/year personally handled		
		1–5	7 cases/20 controls	0.6
		6–20	8 cases/17 controls	0.7
		≥21	1 cases/1 control	1.4
		Year of first use		
		1965 or prior	10 cases/35 controls	0.4
		1966 or later	10 cases/18 controls	1.0
Beane Freeman 2011 [22]	Atrazine	Lifetime days of exposure ^5^		Rate ratio, 95% CI
		Quartile 1, >0–20	41	1.0, Referent
		Quartile 2, 21–56	41	1.1, 0.7–1.7
		Quartile 3, >56–178.5	38	0.9, 0.6–1.5
		Quartile 4, >178.5	32	1.0, 0.6–1.6
				P for trend: 0.7
		Intensity weighted lifetime days of exposure ^5^		
		Quartile 1, >0–20	38	1.0, Referent
		Quartile 2, 21–56	45	1.2, 0.8–1.9
		Quartile 3, >56–178.5	34	0.9, 0.6–1.5
		Quartile 4, >178.5	34	0.9, 0.6–1.5
				P for trend: 0.5
Triazinone herbicides				
Delancey 2009 [29]	Metribuzin	Lifetime days of exposure ^5^		Rate ratio, 95% CI
		No exposure	39	
		Tertile 1, >0–8.75	5	1.0, Referent
		Tertile 2, 8.76–24.5	6	2.3, 0.6–8.6
		Tertile 3, >24.5	8	2.6, 0.8–9.1
				P for trend: 0.1
		Intensity weighted lifetime exposure days ^5^		
		No exposure: 0	39	
		Tertile 1, >0–58.3	4	1.0, Referent
		Tertile 2, >58.3–174.4	6	1.4, 0.3–5.8
		Tertile 3, >174.5	9	2.5, 0.7–9.6
				P for trend: 0.1
**FUNGICIDES**				
McDuffie 2001 [43] ^3^	Captan	Days/year exposed		OR, 95% CI:
		Unexposed	497 cases/1,482 controls	1.0, Referent
		>0–≤2	11 cases/12 controls	2.7, 1.2–6.2
		>2	9 cases/12 controls	2.8, 1.1–6.9
**INSECTICIDES**				
Carbamate insecticides				
Zheng 2001 [60]^ 3^	Carbaryl			OR, 95% CI:
		Non-farmers	243 cases/775 controls	1.0, Referent
		Years since first use		
		<20	19 cases/44 controls	1.1, 0.6–2.0
		≥20	14 cases/21 controls	1.8, 0.9–3.7
		Years of use		
		<7	16 cases/36 controls	1.1, 0.6–2.1
		≥7	15 cases/26 controls	1.5, 0.8–3.0
		Days/year of use		
		<5	9 cases/14 controls	2.4, 1.0–5.9
		≥5	2 cases/4 controls	1.8, 0.3–10.0
Mahajan 2007 [42]	Carbaryl	Lifetime days of exposure ^2^		Rate ratio, 95% CI
		No exposure	23	1.0, Referent
		Tertile 1, 1–9	5	0.7, 0.2–1.8
		Tertile 2, 10–56	8	1.2, 0.5–3.0
		Tertile 3, >56	10	1.7, 0.6–4.5
				P for trend: 0.3
Zheng 2001 [60] ^3^	Carbofuran			OR, 95% CI:
		Nonfarmers	243 cases/775 controls	1.0, Referent
		Years since first use		
		<20	32 cases/63 controls	1.3, 0.8–2.1
		≥20	15 cases/30 controls	1.6, 0.8–3.1
		Years of use		
		<7	30 cases/48 controls	1.7, 1.0–2.9
		≥7	24 cases/47 controls	1.4, 0.8–2.4
		Days/year of use		
		<5	9 cases/15 controls	2.7, 1.1–6.4
		≥5	12 cases/16 controls	3.1, 1.4–6.8
Bonner 2005 [25]	Carbofuran	Lifetime days of exposure ^2^		Rate ratio, 95% CI
		No exposure	44	1.0, Referent
		Tertile 1, 1–9	6	0.8, 0.3–1.9
		Tertile 2, 10–56	7	1.3, 0.6–2.9
		Tertile 3, >56	7	1.4, 0.6–3.3
				P for trend: 0.4
Organophosphorus insecticides				
Fritschi 2005 [62]	Organophosphorus insecticides, group	Degree of exposure ^6^		OR, 95% CI:
		No exposure	662 cases/660 controls	1.0, Referent
		Nonsubstantial exposure	20 cases/28 controls	0.7, 0.4–1.3
		Substantial exposure	12 cases/6 controls	2.1, 0.8–5.7
Waddell 2001 [56] ^3^	Organophosphorus pesticides, group			OR, 95% CI:
		Non-farmers	243 cases/775 controls	1.0, Referent
		Years since first use		
		<20	44 cases/94 controls	1.0, 0.7–1.5
		≥20	79 cases/188 controls	1.6, 1.1–2.2
		Years used		
		<10	34 cases/69 controls	1.1, 0.7–1.7
		10–19	44 cases/71 controls	1.4, 0.9–2.1
		20+	39 cases/59 controls	1.5, 1.0–2.4
Lee 2004 [38]	Chlorpyrifos	Lifetime days of exposure ^1^		Rate ratio, 95% CI:
		Nonexposed	53	1.0, Referent
		Quartile 1, 0.1–8.8	10	0.6, 0.2–1.5
		Quartile 2, 8.9–24.5	13	1.8, 0.9–3.5
		Quartile 3, 24.6–56.0	5	0.9, 0.4–2.4
		Quartile 4, ≥56.1	9	1.0, 0.4–2.4
				P for trend: 0.7
		Intensity-weighted lifetime days of exposure ^1^		
		Nonexposed	53	1.0, Referent
		Quartile 1, 0.1–48.9	6	0.9, 0.3–2.2
		Quartile 2, 49.0–135.9	6	0.6, 0.2–1.8
		Quartile 3, 136.0–417.6	10	1.2, 0.6–2.7
		Quartile 4, ≥417.7	10	1.6, 0.7–3.5
				P for trend: 0.4
Beane Freeman 2005 [21]	Diazinon			Rate ratio, 95% CI:
		No exposure	26	1.0, Referent
		Lifetime days of exposure ^2^		
		Tertile 1, <20	6	1.8, 0.7–4.4
		Tertile 2, 20–38.8	3	1.4, 0.4–4.6
		Tertile 3, >38.8	2	0.9, 0.2–4.1
				P for trend: 1.0
		Intensity-weighted lifetime days of exposure ^2^		
		Tertile 1	5	1.9, 0.7–5.1
		Tertile 2	2	0.7, 0.2–3.1
		Tertile 3	4	1.7, 0.6–5.2
				P for trend: 0.4
Waddell 2001 [56] ^3^	Diazinon			OR, 95% CI:
		Non-farmers	243 cases/775 controls	1.0, Referent
		Years since first use		
		<20	20 cases/34 controls	1.1, 0.6–2.0
		≥20	16 cases/24 controls	1.4, 0.7–2.7
		Years used		
		<10	20 cases/40 controls	0.9, 0.5–1.7
		10–19	10 cases/11 controls	1.8, 0.7–4.4
		20+	1 cases/1 controls	1.9, 0.1–31.6
		Days/year of use		
		<5	6 cases/11 controls	1.3, 0.5–3.9
		≥5	6 cases/6 controls	2.4, 0.7–8.0
Waddell 2001 [56] ^3^	Fonofos			OR, 95% CI:
		Non-farmers	243 cases/775 controls	1.0, Referent
		Years since first use		
		<20	20 cases/36 controls	1.0, 0.6–1.9
		≥20	5 cases/6 controls	1.6, 0.5–5.5
		Years used		
		<10	16 cases/25 controls	1.2, 0.6–2.4
		10–19	7 cases/9 controls	1.5, 0.5–4.1
		20+	2 cases/1 control	4.2, 0.4–47.2
		Days/year of use		
		<5	2 cases/6 controls	0.7, 0.1–3.8
		≥5	9 cases/6 controls	3.4, 1.1–10.3
McDuffie 2001 [43] ^3^	Malathion	Days/year of exposure		OR, 95% CI:
		Unexposed	445 cases/1,379 controls	1.0, Referent
		>0–≤2	50 cases/88 controls	1.8, 1.3–2.7
		≥2	22 cases/39 controls	1.8, 1.0–3.0
Waddell 2001 [56] ^3^	Malathion			OR, 95% CI:
		Non-farmers	243 cases/775 controls	1.0, Referent
		Years since first use		
		<20	22 cases/46 controls	0.9, 0.5–1.6
		≥20	35 cases/39 controls	1.7, 1.1–2.9
		Years used		
		<10	22 cases/39 controls	1.1, 0.6–1.9
		10–19	23 cases/23 controls	1.9, 1.0–3.5
		20+	10 cases/18 controls	1.1, 0.5–2.4
		Days/year of use		
		<5	7 cases/8 controls	2.1, 0.7–6.1
		≥5	5 cases/7 controls	1.5, 0.5–5.2
Bonner 2007 [26]	Malathion	Lifetime days of exposure ^5^		Rate ratio, 95% CI
		No exposure	1.0 Referent	
		Tertile 1, >0–9	0.6, 0.2–1.6	
		Tertile 2, 10–39	0.7, 0.3–1.8	
		Tertile 3, >39	0.8, 0.3–2.0	
		Intensity-weighted lifetime days of exposure ^2^		Rate ratio, 95% CI
		No exposure	14	1.0, Referent
		Tertile 1, >0–58	5	0.5, 0.2–1.5
		Tertile 2, 59–245	9	0.7, 0.3–1.8
		Tertile 3, >245	9	0.8, 0.3–2.0
				P for trend: 0.9
Waddell 2001 [56] ^3^	Phorate			OR, 95% CI:
		Non-farmers	243 cases/775 controls	1.0, Referent
		Years since first use		
		<20	19 cases/43 controls	0.8, 0.4–1.5
		≥20	14 cases/23 controls	1.3, 0.6–2.6
		Years used		
		<10	20 cases/33 controls	1.2, 0.6–2.1
		10–19	9 cases/19 controls	0.9, 0.4–2.1
		20+	4 cases/5 controls	1.5, 0.4–5.9
		Days/year of use		
		<5 days	5 cases/9 controls	1.3, 0.4–4.0
		≥5 days	7 cases/8 controls	2.0, 0.7–5.9
Waddell 2001 [56] ^3^	Terbufos			OR, 95% CI:
		Non-farmers	243 cases/775 controls	1.0, Referent
		Years since first use		
		<20	23 cases/51 controls	0.9, 0.5-1.5
		≥20	0 cases/1 control	-
		Years used		
		<10	13 cases/38 controls	0.6, 0.3–1.3
		10–19	6 cases/8 controls	1.5, 0.5–4.4
		20+	0 cases/1 control	-
		<20	23 cases/51 controls	0.9, 0.5–1.5
		≥20	0 cases/1 control	-
		Days/year of use		
		<5	3 cases/8 controls	0.8, 0.2–3.4
		≥5	7 cases/4 controls	4.0, 1.1–14.5
Bonner 2010 [24]	Terbufos	Intensity weighted lifetime exposure days ^5^		Hazard ratio, 95% CI
		No exposure	69	1.0, referent
		Tertile 1, >0–107	17	1.3, 0.7–2.3
		Tertile 2, >107–352	24	1.9, 1.2–3.2
		Tertile 3, >352	15	1.2, 0.7–2.2
				P for trend: 0.6
Organochlorine insecticides				
Fritschi 2005 [62]	Organochlorines, group	Degree of exposure ^6^		OR, 95% CI:
		None	674 cases/679 controls	1.0, Referent
		Nonsubstantial	14 cases/13 controls	1.1, 0.5–2.3
		Substantial	6 cases/2 controls	3.3, 0.7–16.4
Purdue 2007 [51] ^3^	Organochlorines, group	Lifetime days of exposure ^5^		Rate ratio, 95% CI:
		Unexposed	16	1.0, Referent
		Tertile 1, 1–110	8	1.2, 0.5–2.8
		Tertile 2, 111–450	10	1.5, 0.6–3.5
		Tertile 3, >450	11	1.5, 0.6–3.8
				P for trend: 0.3
		Intensity weighted lifetime days of exposure ^5^		
		Unexposed	16	1.0, Referent
		Tertile 1, 1–110	9	1.3, 0.6–3.1
		Tertile 2, 111–450	7	1.1, 0.4–2.9
		Tertile 3, >450	13	1.7, 0.7–4.2
				P for trend: 0.3
Purdue 2007 [51] ^3^	Aldrin	Lifetime days of exposure ^5^		Rate ratio, 95% CI:
		Unexposed	38	1.0, Referent
		1–20	5	0.8, 0.3–2.1
		>20	4	0.4, 0.1–1.5
				P for trend: 0.2
		Intensity weighted lifetime days of exposure ^5^		
		Unexposed	38	1.0, Referent
		1–20	4	0.6, 0.2–1.9
		>20	5	0.6, 0.2–1.8
				P for trend: 0.4
Purdue 2007 [51] ^3^	Chlordane	Lifetime days of exposure ^5^		Rate ratio, 95% CI:
		Unexposed	32	1.0, Referent
		1–9	9	1.6, 0.8–3.6
		>9	6	1.8, 0.7–4.6
				P for trend: 0.2
		Intensity weighted lifetime days of exposure ^5^		
		Unexposed	32	1.0, Referent
		1–9	8	1.8, 0.8–4.0
		>9	7	1.6, 0.7–3.9
				P for trend: 0.3
Baris 1998 [20] ^3^	DDT			OR, 95% CI:
		Non-farmers	243 cases/775 controls	1.0, Referent
		Days/year of use		
		≤5	12 cases/35 controls	1.0, 0.5–2.1
		>5	11 cases/15 controls	2.1, 0.9–4.9
		Duration of use in years		
		1–4	36 cases/79 controls	1.0, 0.7–1.6
		5–9	31 cases/53 controls	1.4, 0.8–2.2
		≥15	39 cases/64 controls	1.5, 0.9–2.3
McDuffie 2001 [43] ^3^	DDT	Days/year of exposure		OR, 95% CI:
		Unexposed	485 cases/1,447 controls	1.0, Referent
		>0–≤2	18 cases/32 controls	1.8, 1.0–3.2
		≥2	14 cases/27 controls	1.5, 0.8–2.9
Hardell 2002 [33] ^3^	DDT			OR, 95% CI:
		Never exposed	NR	1.0, Referent
		Years between first exposure and diagnosis		
		1–10	NR	-
		>10–20	NR	2.6, 0.6–11.0
		>20–30	NR	1.6, 0.8–3.3
		>30	NR	1.2, 0.8–1.7
		Years between last exposure and diagnosis		
		1–10	NR	1.5, 0.7–3.1
		>10–20	NR	1.1, 0.6–2.0
		>20–30	NR	1.5, 0.8–2.5
		>30	NR	1.2, 0.7–2.0
Purdue 2007 [51] ^3^	DDT			
		Lifetime days of exposure ^5^		Rate ratio, 95% CI:
		Unexposed	32	1.0, Referent
		1–20	5	0.7, 0.3–1.9
		>20	9	1.2, 0.5–2.8
				P for trend: 0.6
		Intensity weighted lifetime days of exposure ^5^		
		Unexposed	32	1.0, Referent
		1–20	6	0.9, 0.3–2.2
		>20	8	1.0, 0.4–2.5
				P for trend: 0.9
Eriksson 2008 [32] ^3^	DDT	Days of exposure ^4^		
		≤37 days	20 cases/19 controls	1.2, 0.6–2.2
		>37 days	30 cases/18 controls	1.8, 1.0–3.2
Purdue 2007 [51] ^3^	Dieldrin			
		Lifetime days of exposure ^5^		Rate ratio, 95% CI:
		Unexposed	46	1.0, Referent
		1–20	1	0.6, 0.1–4.2
		>20	1	0.9, 0.1–6.9
				P for trend: 0.8
		Intensity weighted lifetime days of exposure ^5^		
		Unexposed	46	1.0, Referent
		1–20	1	0.7, 0.1–5.0
		>20	1	0.7, 0.1–5.5
				P for trend: 0.7
Purdue 2007 [51] ^3^	Heptachlor	Lifetime days of exposure		Rate ratio, 95% CI:
		Unexposed	38	1.0, Referent
		1–9	6	1.5, 0.6–4.1
		>9	4	1.1, 0.4–3.2
				P for trend: 0.8
		Intensity weighted lifetime days of exposure		
		Unexposed	38	1.0, Referent
		1–9	4	1.2, 0.4–3.6
		>9	6	1.4, 0.5–3.7
				P for trend:0.6
Blair 1998 [23] ^3^	Lindane			OR, 95% CI:
		Nonfarmer	243 cases/775 controls	1.0, Referent
		Years since first use		
		≥20	59 cases/83 controls	1.7, 1.1–2.5
		<20	18 cases/30 controls	1.3, 0.7–2.4
		Days/ year of use		
		≤4	8 cases/16 controls	1.6, 0.6–4.0
		≥5	5 cases/8 controls	2.0, 0.6–6.4
Rafnsson 2006 [52] ^3^	Lindane	Number of sheep owned		OR, 95% CI:
		100–199 sheep	22 cases/71 controls	3.8, 1.6–9.3
		200–683 sheep	15 cases/62 controls	3.4, 1.3–9.0
Purdue 2007 [51] ^3^	Lindane	Lifetime days exposed ^5^		Rate ratio, 95% CI:
		Unexposed	34	1.0, Referent
		1–22 days	6	1.9, 0.8–4.7
		>22 days	7	2.1, 0.8–5.5
				P for trend: 0.1
		Intensity-weighted lifetime days of exposure ^5^		
		Unexposed	34	1.0, Referent
		1–22	5	0.9, 0.3–3.0
		>22	8	2.6, 1.1–6.4
				P for trend: 0.04
Purdue 2007 [51] ^3^	Toxaphene	Lifetime days exposed ^5^		Rate ratio, 95% CI:
		Unexposed	35	1.0, Referent
		1–25 days	10	2.3, 1.1–5.0
		>25 days	2	1.3, 0.3–5.5
				P for trend: 0.8
		Intensity-weighted lifetime days of exposure ^5^		
		Unexposed	35	1.0, Referent
		1–25	7	2.3, 1.0–5.4
		>25	5	1.6, 0.5–4.8
				P for trend:0.4
Pyrethroid insecticides				
Rusiecki 2009 [53]	Permethrin	Lifetime days of exposure ^5^		Relative rate, 95% CI:
		Nonexposed	94	1.0, Referent
		Tertile 1, ≤8.74	8	0.8, 0.4–1.7
		Tertile 2, 8.75–50.75	5	0.6, 0.3–1.7
		Tertile 3, >50.75	5	0.7, 0.3–1.7
				P for trend: 0.2
		Intensity-weighted lifetime days of exposure ^5^		
		Nonexposed	94	1.0, Referent
		Tertile 1, ≤8.74	7	0.8, 0.4–1.8
		Tertile 2, 8.75–50.75	7	0.9, 0.4–2.0
		Tertile 3, >50.75	4	0.5, 0.2–1.3
				P for trend: 0.2
Other insecticides				
Eriksson 2008 [32] ^3^	Pyretrine	Days of exposure ^4^		OR, 95% CI:
		≤25	8 cases/5 controls	1.9, 0.6–5.8
		>25	6 cases/5 controls	1.4, 0.4–4.5
	Mercurial seed dressing	Days of exposure ^4^		
		≤12	7 cases/6 controls	1.3, 0.4–3.8
		>12	14 cases/5 controls	2.9, 1.0–8.3
	Fumigant fungicides			
Barry 2012 [19]	Methyl Bromide	Intensity weighted lifetime days of exposure ^5^		Rate ratio, 95% CI:
		Nonexposed	166	1.0, Referent
		Tertile 1, >0–310	21	2.3, 1.4–3.9
		Tertile 2, 311–1519	8	0.7, 0.3–1.6
		Tertile 3, >1519	6	0.6, 0.3–1.5
				P for trend: 0.1
		Intensity weighted lifetime days of exposure, 15 year lag ^5^		
		Nonexposed	174	1.0, Referent
		Tertile 1, >0–310	13	1.7, 0.9–3.1
		Tertile 2, 311–1519	6	0.6, 0.3–1.5
		Tertile 3, >1519	8	1.0, 0.5–2.1
				P for trend: 0.7

Notes: CI, confidence interval; EPTC, s-ethyl dipropylthiocarbamate; MCPA, 2-methyl-4-chlorophenoxyacetic acid; NHL, non-Hodgkin lymphoma; OR, odds ratio; CI, confidence interval; **^1^** Categories based on mid-points of the questionnaire category; **^2^** Categories based on distribution among users; **^3^** Effect estimates in association with dichotomous exposure were also reported; **^4^** Categories based on the number of days of exposure among controls; **^5^** Categories based on the distribution of exposure among cancer cases; **^6^** Substantial indicates the person was exposed to the substance at a medium or high level for more than five 8-hour days per year for a combined total of more than 5 years. Non-substantial indicates any other combination of exposures; estimates derive from a case-control study.

**Table 3:** Associations of subtypes of non-Hodgkin lymphoma with herbicides and insecticides.

	Chemical	Number of exposed cases	Risk ratio, 95% CI
**B cell lymphoma**			
***HERBICIDES***			
*Organophosphorus herbicides*			
Eriksson 2008 [32]	Glyphosate (OP herbicide)	NR	1.9, 1.0–3.5
*Phenoxy herbicides*			
Cocco 2012 [28]	Phenoxy herbicides	12 cases	1.4, 0.6–3.1
Eriksson 2008 [32]	Phenoxy herbicides	NR	2.0, 1.2–3.3
Fritschi 2005 [62] ^1^	Phenoxy herbicides	NR	No exposure: 1.0, Referent
			Non-substantial exposure: 0.6, 0.3–1.5
			Substantial exposure: 1.5, 0.3–6.6
Eriksson 2008 [32]	2,4,5-T and/or 2,4-D (Phenoxy herbicide)	NR	1.7, 0.9–3.0
Cocco 2012 [28]	2,4-D (Phenoxy herbicide)	2 cases	0.6, 0.1–3.5
Cocco 2012 [28]	MCPA (Phenoxy herbicide)	4 cases	Infinity (zero exposed controls)
Eriksson 2008 [32]	MCPA (Phenoxy herbicide)	NR	2.6, 1.1–5.9
*Thiocarbamate herbicides*			
Zheng 2001 [60]	Butylate (Thiocarbamate herbicides)	4 cases (small lymphocytic)	1.1, 0.3–3.4
Zheng 2001 [60]	EPTC + Protectant (Thiocarbamate herbicides)	2 cases (small lymphocytic)	1.5, 0.3–7.1
Cocco 2012 [28]	Triazines and triazoles	6 cases	0.7, 0.2–1.7
***INSECTICIDES***			
*Carbamate insecticides*			
Zheng 2001 [60]	Carbaryl (Carbamate insecticide)	9 cases(small lymphocytic)	2.9, 1.2–7.0
Zheng 2001 [60]	Carbofuran (Carbamate insecticide)	7 cases (small lymphocytic)	1.5, 0.6–3.8
Cocco 2012 [28]	Methomyl (Carbamate insecticide)	0 cases	NR(zero exposed cases)
Cocco 2012 [28]	Mancozeb (Dithiocarbate fungicide)	2 cases	0.6, 0.1–3.5
Cocco 2012 [28]	Glyphosate (OP herbicide)	4 cases	3.1, 0.6–17.1
*Organochlorine (OC) insecticides*			
Cocco 2012 [28]	Organochlorines	27 cases	0.9, 0.5–1.4
Fritschi 2005 [62]^ 1^	Organochlorines	NR	No exposure: 1.0, Referent
			Nonsubstantial: 1.1, 0.5–2.5
			Substantial: 3.5, 0.7–17.3
Baris 1998 [20] ^2^	DDT (OC insecticides)	22 cases	1.6, 0.8–2.9
Eriksson 2008 [32]	DDT (OC insecticide)	NR	1.3, 0.8–2.1
Cocco 2012 [28]	DDT (OC insecticide)	3 cases	1.2, 0.2–5.9
Cocco 2012 [28]	Endosulfan (OC insecticide)	0 cases	NR, zero exposed cases
*Organophosphorus insecticides*			
Cocco 2012 [28]	Organophosphates	23 cases	1.4, 0.8, 2.6
Zheng 2001 [60]^ 2^	Organophosphates	18 cases	1.6, 0.8–3.2
Fritschi 2005 [62] ^1^	Organophosphates	NR	No exposure ^1^: 1.0, Referent
			Non-substantial: 0.6, 0.3–1.2
			Substantial: 2.1, 0.8–5.7
Cocco 2012 [28]	Dimethoate (OP insecticide)	3 cases	1.8, 0.3–10.6
Waddell 2001 [56]^ 2^	Fonofos (OP insecticide)	5 cases	2.6, 0.8–8.5
Waddell 2001 [56]^ 2^	Malathion (OP insecticide)	10 cases	1.9, 0.8–4.7
Waddell 2001 [56]^ 2^	Diazinon (OP insecticide)	9 cases	2.8, 1.1–7.3
Waddell 2001 [56]^ 2^	Phorate (OP insecticides)	8 cases	2.3, 0.9–6.0
Waddell 2001 [56]^ 2^	Terbufos (OP insecticides)	5 cases	2.2, 0.7–7.4
*Other insecticides*			
Eriksson 2008 [32]	Pyrethrine (Botanical insecticide)	NR	1.7, 0.7–3.9
Eriksson 2008 [32]	Mercurial seed dressing	NR	1.8, 0.8–3.9
**Mature B cell lymphoma**			
Beane Freeman 2011 [22]	Atrazine (Triazine herbicide)	Lifetime days of exposure:	
		Quartile 1, >0–20: 36	1.0, Referent
		Quartile 2, 21–56: 34	1.0, 0.7–1.7
		Quartile 3, >56–178.5: 31	0.9, 0.5–1.4
		Quartile 4, >178.5: 28	0.9, 0.6–1.6
			P for trend: 0.8
		Intensity weighted lifetime days of exposure:	
		Quartile 1, >0–20: 34	1.0, Referent
		Quartile 2, 21–56: 38	1.1, 0.7–1.8
		Quartile 3, >56–178.5: 25	0.8, 0.5–1.3
		Quartile 4, >178.5: 31	0.9, 0.6, 1.5
			P for trend: 0.7
**Diffuse large B cell lymphoma**			
***HERBICIDES***			
*Organophosphorus herbicides*			
Eriksson 2008 [32]	Glyphosate (OP herbicides)	NR	1.2, 0.4–3.4
*Phenoxy herbicides*			
Cocco 2012 [28]	Phenoxy herbicides	4 cases	1.7, 0.5–5.2
Eriksson 2008 [32]	Phenoxy herbicides	NR	2.2, 1.1–4.3
Fritschi 2005 [62] ^1^	Phenoxy herbicides	NR	No exposure ^1^: 1.0, Referent
			Nonsubstantial exposure: 0.5, 0.1–2.0
			Substantial exposure: 2.2, 0.4–13.1
Eriksson 2008 [32]	MCPA (Phenoxy herbicide)	NR	3.9, 1.5–10.5
Eriksson 2008 [32]	2,4,5-T and/or 2,4-D (Phenoxy herbicide)	NR	1.7, 0.7–3.8
*Thiocarbamate herbicides*			
Zheng 2001 [60]	Butylate (Thiocarbamate herbicides)	15 cases	1.6, 0.9–3.1
Zheng 2001 [60]	EPTC + Protectant (Thiocarbamate herbicides)	10 cases	1.8, 0.8–3.7
*Triazine herbicides*			
Cocco 2012 [28]	Triazines and triazoles	2 cases	0.8, 0.2–3.4
Beane Freeman 2011 [22]	Atrazine (Triazine herbicides)	Lifetime exposure days:	
		Quartile 1, >0–20: 20	1.0, Referent
		Quartile 2, 21–56: 14	0.8, 0.4–1.6
		Quartile 3, >56–178.5: 14	0.7, 0.4–1.5
		Quartile 4, >178.5: 11	0.7, 0.3–1.6
			p for trend:0.5
		Intensity-weighted lifetime exposure days:	
		Quartile 1, >0–20: 15	1.0, Referent
		Quartile 2, 21–56: 18	1.2, 0.6–2.5
		Quartile 3, >56–178.5: 11	0.8, 0.4–1.7
		Quartile 4, >178.5: 15	1.1, 0.5–2.3
			p for trend:0.96
Zahm 1993 [59]	Atrazine (Triazine herbicides)	66 cases	1.6, 1.1–2.2
***INSECTICIDES***			
*Carbamate insecticides*			
Zheng 2001 [60]	Carbaryl (Carbamate insecticides)	15 cases	1.5, 0.8–2.8
Zheng 2001 [60]	Carbofuran (Carbamate insecticides)	24 cases	1.6, 1.0–2.7
*Organochlorine insecticides*			
Cocco 2012 [28]	Organochlorines	5 cases	0.6, 0.2–1.6
Fritschi 2005 [62]^ 1^	Organochlorines	NR	No exposure ^1^: 1.0, Referent
			Non-substantial exposure: 1.2, 0.4–3.4
			Substantial exposure: 1.6, 0.2–18.1
Eriksson 2008 [32]	DDT (OC insecticide)	NR	1.2, 0.6–2.5
Baris 1998 [20]	DDT (OC insecticide)	53 cases	1.2, 0.8–1.7
*Organophosphorus insecticides*			
Cocco 2012 [28]	Organophosphates	5 cases	1.1, 0.4–2.9
Waddell 2001 [56]	Organophosphates	63 cases	1.8, 1.2–2.6
Fritschi 2005 [62]	Organophosphates	NR	No exposure ^1^: 1.0, Referent
			Non-substantial exposure: 0.6, 0.3–1.6
			Substantial exposure: 2.1, 0.6–7.7
Waddell 2001 [56]	Fonofos (OP insecticide)	10 cases	1.3, 0.6–2.7
Waddell 2001 [56]	Malathion (OP insecticide)	19 cases	1.1, 0.6–1.9
Waddell 2001 [56]	Diazinon (OP insecticide)	13 cases	1.2, 0.6–2.4
Waddell 2001 [56]	Phorate (OP insecticide)	10 cases	0.8, 0.4–1.8
Waddell 2001 [56]	Terbufos (OP insecticide)	7 cases	0.8, 0.4–2.0
*Other insecticides*			
Cocco 2012 [28]	Arsenicals	2 cases	0.4, 0.1–1.6
Eriksson 2008 [32]	Pyrethrine (Botanical insecticide)	NR	1.3, 0.3–4.6
Eriksson 2008 [32]	Mercurial seed dressing	NR	2.2, 0.8–6.1
**Chronic lymphocytic leukemia**			
***HERBICIDES***			
Cocco 2012 [28]	Phenoxy acids	Ever *vs.* never exposed	
		2 cases ever exposed	0.9, 0.2–4.1
		Intensity of exposure	
		Unexposed: 362 cases	1.0, Referent
		Low: 0 cases	
		Medium/high: 2 cases	2.4, 0.4–13.8
Cocco 2012 [28]	Triazines and triazoles	2 cases	0.9, 0.2–4.1
***INSECTICIDES***			
Cocco 2012 [28]	Arsenicals	15 cases	1.6, 0.8–2.9
Cocco 2012 [28]	Carbamates		
Cocco 2012 [28]	Organochlorines	Ever *vs* never exposed	
		10 cases ever exposed	1.2, 0.6–2.5
		Intensity of exposure	
		Unexposed: 362 cases	1.0, Referent
		Low: 5 cases	1.8, 0.6–5.0
		Medium/high: 5 cases	1.0, 0.4–2.8
Cocco 2012 [28]	Organophosphates	Ever *vs* never exposed	
		9 cases ever exposed	2.7, 1.2–6.0
		Intensity of exposure	
		Unexposed: 362 cases	1.0, Referent
		Low: 5 cases	2.7, 0.9–7.8
		Medium/high: 4 cases	2.6, 0.7–9.2
**Lymphocytic lymphoma**			
***HERBICIDES***			
*Organophosphorus herbicides*			
Eriksson 2008 [32]	Glyphosate (OP herbicide)	NR	3.4, 1.4–7.9
*Phenoxy herbicides*			
Eriksson 2008 [32]	Phenoxy herbicides	NR	2.1, 1.0–4.5
Cocco 2013 [28]	Phenoxy herbicides	NR	0.9, 0.2–4.1
Eriksson 2008 [32]	2,4,5-T and/or 2,4-D (Phenoxy herbicides)	NR	1.9, 0.9–4.4
Eriksson 2008 [32]	MCPA (Phenoxy herbicides)	NR	2.6, 0.7–9.0
***INSECTICIDES***			
*Organochlorine insecticides*			
Eriksson 2008 [32]	DDT (OC insecticides)	NR	1.4, 0.7–2.8
*Organophosphorus insecticides*			
Fritschi 2005 [62]^ 1^	Organophosphates	NR	No exposure: 1.0, Referent
			Non-substantial exposure: 1.1, 0.5–2.3
			Substantial exposure: 4.3, 1.4–13.0
*Other insecticides*			
Eriksson 2008 [32]	Pyrethrine (Botanical insecticide)	NR	2.4, 0.7–7.9
Eriksson 2008 [32]	Mercurial seed dressing	NR	2.9, 1.0–8.3
**Follicular lymphoma**			
***HERBICIDES***			
*Organophosphorus herbicides*			
Eriksson 2008 [32]	Glyphosate (OP herbicide)	NR	1.9, 0.6–5.8
*Phenoxy herbicides*			
Eriksson 2008 [32]	Phenoxy herbicides	NR	1.3, 0.4–3.8
Fritschi 2005 [62]^ 1^	Phenoxy herbicides	NR	No exposure: 1.0, Referent
			Non-substantial exposure: 0.5, 0.1–2.0
			Substantial exposure: 1.2, 0.1–11.2
Eriksson 2008 [32]	2,4,5-T and/or 2,4-D (Phenoxy herbicide)	NR	1.2, 0.4–4.2
Eriksson 2008 [32]	MCPA (Phenoxy herbicide)	NR	No exposed cases
*Thiocarbamate herbicides*			
Zheng 2001 [60]	Butylate (Thiocarbamate herbicides)	17 cases	1.5, 0.8–2.8
Zheng 2001 [60]	EPTC + Protectant use (Thiocarbamate herbicides)	10 cases	1.7, 0.8–3.8
*Triazine herbicides*			
Zahm 1993 [59]	Atrazine (Triazine herbicide)	40 cases	1.3, 0.9–1.9
Beane Freeman 2011 [22]	Atrazine (Triazine herbicide)	Lifetime exposure days, by quartile:	
		Quartile 1, >0–20: 10	1.0, Referent
		Quartile 2, 21–56: 8	0.9, 0.3–2.2
		Quartile 3, >56–178.5: 6	0.6, 0.2–1.7
		Quartile 4, >178.5: 8	1.0, 0.4–2.6
			p for trend: 0.9
		Intensity-weighted exposure days:	
		Quartile 1, >0–20: 10	1.0, Referent
		Quartile 2, 21–56: 10	1.0, 0.4–2.4
		Quartile 3, >56–178.5: 8	0.8, 0.3–2.1
		Quartile 4, >178.5: 4	0.4, 0.1–1.3
			p for trend: 0.07
***INSECTICIDES***			
*Carbamate insecticides*			
Zheng 2001 [60]	Carbaryl (Carbamate insecticides)	14 cases	1.3, 0.6–2.4
Zheng 2001 [60]	Carbofuran (Carbamate insecticides)	22 cases	1.4, 0.8–2.4
*Organochlorine insecticides*			
Fritschi 2005 [62]^ 1^	Organochlorines	NR	No exposure: 1.0, Referent
			Non-substantial exposure: 1.8, 0.7–4.8
			Substantial exposure: 3.5, 0.5–25.2
Eriksson 2008 [32]	DDT (OC insecticide)	NR	2.1, 1.1–4.4
Baris 1998 [20]	DDT (OC insecticide)	47 cases	1.3, 0.8–1.9
*Organophosphorus insecticides*			
Waddell 2001 [56]	OP pesticides, group	50 cases	1.3, 0.9–2.0
Waddell 2001 [56]	Fonofos (OP insecticide)	14 cases	1.2, 0.6–2.4
Waddell 2001 [56]	Malathion (OP insecticide)	29 cases	1.3, 0.8–2.2
Waddell 2001 [56]	Diazinon (OP insecticide)	17 cases	1.3, 0.7–2.3
Waddell 2001 [56]	Phorate (OP insecticide)	10 cases	0.7, 0.3–1.4
Waddell 2001 [56]	Terbufos (OP insecticide)	9 cases	0.7, 0.3–1.6
Eriksson 2008 [32]	Mercurial seed dressing	NR	3.6, 1.2–10.9
Eriksson 2008 [32]	Pyrethrine (Botanical insecticide)	NR	2.6, 0.8–8.5
**T cell lymphoma**			
***HERBICIDES***			
*Organophosphorus herbicides*			
Eriksson 2008 [32]	Glyphosate (OP insecticide)	NR	2.3, 0.5–10.4
*Phenoxy herbicides*			
Eriksson 2008 [32]	Phenoxy herbicides	NR	1.6, 0.4–7.3
Eriksson 2008 [32]	2,4,5-T and/or 2,4-D (Phenoxy herbicides)	NR	1.0, 0.1–8.0
Eriksson 2008 [32]	MCPA (Phenoxy herbicides)	NR	2.4, 0.3–20.0
***INSECTICIDES***			
Eriksson 2008 [32]	DDT (OC insecticide)	NR	2.9, 1.1–8.0
Eriksson 2008 [32]	Mercurial seed dressing	NR	2.1, 0.3–17.1
Eriksson 2008 [32]	Pyrethrine (Botanical insecticide)	NR	2.2, 0.3–17.8
***Unspecified NHL***			
***HERBICIDES***			
*Organophosphorus herbicides*			
Eriksson 2008 [32]	Glyphosate (OP insecticide)	NR	5.6, 1.4–22.0
*Phenoxy herbicides*			
Eriksson 2009 [32]	Phenoxy herbicides	NR	3.8, 1.2–12.1
Eriksson 2008 [32]	2,4,5-T and/or 2,4-D (Phenoxy herbicide)	NR	3.2, 0.9–12.1
Eriksson 2008 [32]	MCPA (Phenoxy herbicide)	NR	9.3, 2.1–41.2
***INSECTICIDES***			
Eriksson 2008 [32]	DDT (OC insecticide)	NR	2.4, 0.8–7.4
Eriksson 2008 [32]	Mercurial seed dressing	NR	5.4, 1.3–22.0
Eriksson 2008 [32]	Pyrethrine (Botanical insecticide)	NR	3.1, 0.4–26.3
**Other specified B cell lymphoma**			
***HERBICIDES***			
*Organophosphorus herbicides*			
Eriksson 2008 [32]	Glyphosate (OP herbicide)	NR	1.6, 0.5–5.0
*Phenoxy herbicides*			
Eriksson 2008 [32]	Phenoxy herbicides	NR	2.6, 1.2–5.6
Eriksson 2008 [32]	2,4,5-T and/or 2,4-D (Phenoxy herbicide)	NR	2.2, 0.9–5.4
Eriksson 2008 [32]	MCPA (Phenoxy herbicide)	NR	3.2, 1.0–10.7
***INSECTICIDES***			
Eriksson 2008 [32]	DDT (OC insecticide)	NR	1.3, 0.6–3.1
Eriksson 2008 [32]	Mercurial seed dressing	NR	2.4, 0.7–7.8
Eriksson 2008 [32]	Pyretrine	NR	1.5, 0.3–6.9
**Unspecified B cell lymphoma**			
***HERBICIDES***			
*Organophosphorus herbicides*			
Eriksson 2008 [32]	Glyphosate (OP herbicide)	NR	1.5, 0.3–6.6
*Phenoxy herbicides*			
Eriksson 2008 [32]	Phenoxy herbicides	NR	1.1, 0.3–4.0
Eriksson 2008 [32]	2,4,5-T and/or 2,4-D (Phenoxy herbicide)	NR	0.9, 0.2–3.9
Eriksson 2008 [32]	MCPA (Phenoxy herbicide)	NR	1.4, 0.2–11.2
***INSECTICIDES***			
Eriksson 2008 [32]	DDT (OC insecticide)	NR	0.2, 0.0–1.8
Eriksson 2008 [32]	Mercurial seed dressing	NR	No exposed cases

Notes: 2,4-D, 2,4-Dichlorophenoxyacetic acid; 2,4,5-T, 2,4,5-Trichlorophenoxyacetic acid; DDT, dichlorodiphenyltrichloroethane; EPTC, s-ethyl dipropylthiocarbamate; MCPA, 2-methyl-4-chlorophenoxyacetic acid; NHL, non-Hodgkin lymphoma; NR, Not reported; OC, Organochlorine; OP, Organophosphorus; **^1^** Substantial indicates the person was exposed to the substance at a medium or high level for more than five 8-hour days per year for a combined total of more than 5 years. Nonsubstantial indicates any other combination of exposures; estimates derive from a case-control study; **^2^** NHL subtype is labeled small lymphocytic in the paper.

In the Agricultural Health Study, Delancey *et al.* [29] observed a fairly strong dose response relationship between exposure to metribuzin, a triazinone herbicide, and NHL (P for trend: 0.13). Waddell *et al.* [56] observed a dose-response relationship between years of use of the organophosphorus insecticide fonofos and NHL. These authors also observed a strong positive relationship between days/year of exposure to another organophosphorus insecticide, terbufos, and NHL (OR, 95% CI for ≥5 days *vs.* non-farmers: 4.0, 1.1–14.5). 

Table 3 shows estimates of association between subtypes of NHL and chemical groups or active ingredients. Table 4 shows the individual effect estimates of associations with herbicides, fungicides, and insecticides, coded dichotomously. 

**Table 4:** Effect estimates from papers that investigated associations between non-Hodgkin lymphoma and herbicide, fungicide, and insecticide exposures, categorized dichotomously.

Author, date	N exposed	Risk ratio, 95% CI
**HERBICIDES**		
**Amide herbicides**		
*Amide herbicides, group*		
Hoar 1986 [34]	8 cases/22 controls	2.9, 1.1–7.6
Cantor 1992 [27]	58 cases/114 controls	1.2, 0.8–1.7
Zahm 1993 [18] ^1^	8 cases/34 controls	0.9, 0.4–2.2
Orsi 2009 [46]	5 cases/12 controls	0.9, 0.3–2.8
*Alachlor*		
De Roos 2003 [30]	68 cases/152 controls	1.1, 0.7–1.7
Lee 2004 [39] ^2^	29 cases	0.7, 0.5–1.1
*Metolachlor*		
De Roos 2003 [30]	13 cases/37 controls	0.7, 0.3–1.6
*Propachlor*		
De Roos 2003 [30]	20 cases/50 controls	1.0, 0.5–2.0
*Propyzamide*		
Mills 2005 [45]	NR	0.7, 0.3–1.4
**Organophosphorus herbicides**		
*Glyphosate*		
McDuffie 2001 [43]	51 cases/133 controls	1.2, 0.8–1.7
Hardell 2002 [33]	8 cases/8 controls	3.0, 1.1–8.5
De Roos 2003 [30]	36 cases/61 controls	2.1, 1.1–4.0
De Roos 2005 [31] ^2^	71 cases	1.1, 0.7–1.9
Eriksson 2008 [32]	29 cases/18 controls	2.0, 1.1–3.7
Orsi 2009 [46]	12 cases/24controls	1.0, 0.5–2.2
*Phosphonic acid*		
McDuffie 2001 [43]	63 cases/147 controls	1.4, 0.9–1.9
**Phenoxy herbicides**		
*Phenoxy herbicides, group*		
Hoar 1986 [34]	24 cases/78 controls	2.2, 1.2–4.1
Pearce 1987 [48]	81 cases/143 controls	1.0, 0.8–1.4
Woods 1987 [57]	NR	1.3, 0.9–2.0
Persson 1989 [49]	6 cases/6 controls	4.9, 1.0–23.5
Cantor 1992 [27]	118 cases/231 controls	1.2, 0.9–1.6
Persson 1993 [50]	10 cases/14 controls	2.3, 0.2–2.8
Zahm 1993 [18]^ 1^	14 cases/63 controls	0.9, 0.4–1.7
Hardell 2002 [33]	64 cases/90 controls	1.7, 1.2–2.3
Miligi 2006 [44]	32 cases/28 controls	1.1, 0.6–1.8
Eriksson 2008 [32]	47 cases/26 controls	2.0, 1.2–3.4
Orsi 2009 [46]	11 cases/25 controls	0.9, 0.4–1.9
Pahwa 2012 [47]	129 cases/138 controls	1.5, 1.1–1.9
*2,4-D*		
Zahm 1990 [58]	43 cases/98 controls	1.5, 0.9–2.5
Cantor 1992 [27]	Ever handled: 115 cases/227 controls	1.2, 0.9–1.6
Cantor 1992 [27] ^3^	Handled prior to 1965: 86 cases/153 controls	1.3, 0.9–1.8
Mills 2005 [45]	NR	3.8, 1.9–7.8
Miligi 2006 [44]	17 cases/18 controls	0.9, 0.5–1.8
Pahwa 2012 [47]	110 cases/293 controls	1.3, 1.0–1.7
*2,4,5-T*		
De Roos 2003 [30]	Ever handled: 25 cases/63 controls	1.0, 0.5–1.9
Cantor 1992 [27] ^3^	Handled prior to 1965: 13 cases/18 controls	1.7, 0.8–3.6
*2,4,5-T and/or 2,4-D*		
Eriksson 2008 [32]	33 cases/21 controls	1.6, 0.9–3.0
*Diclofop-methyl*		
McDuffie 2001 [43]	9 cases/25 controls	1.0, 0.4–2.2
*MCPA*		
Hardell 2002 [33]	21 cases/23 controls	2.6, 1.4–4.9
De Roos 2003 [30] ^1^	8 cases/16 controls	1.0, 0.4–2.6
Miligi 2006 [44]	18 cases/19 controls	0.9, 0.4–1.8
Eriksson 2008 [32]	21 cases/9 controls	2.8, 1.3–6.2
Pahwa 2012 [47]	17 cases/46 controls	1.1, 0.6–2.0
**Carbamate/Thiocarbamate herbicides**		
*Carbamate/Thiocarbamate herbicides, group*		
Zahm 1993 [18] ^1^	2 cases/14 controls	0.6, 0.1–2.8
McDuffie 2001 [43]	21 cases/49 controls	1.5, 0.8–2.6
Zheng 2001 [60]	60 cases/108 controls	1.5, 1.1–2.3
*Butylate*		
Cantor 1992 [27] ^3^	Handled prior to 1965: 1 case/6 controls	0.5, 0.1–4.3
Zheng 2001 [60]	45 cases/76 controls	1.6, 1.0–2.4
*Diallate*		
McDuffie 2001 [43]	11 cases/29 controls	1.5, 0.7–3.1
*EPTC + Protectant*		
Zheng 2001 [60]	23 cases/49 controls	1.6, 0.9–2.7
**Aromatic acid herbicides**		
*Benzoic acid herbicides*		
Hoar 1986 [34]	1 case/2 controls	4.0, 0.1–62.6
Cantor 1992 [27]	53 cases/98 controls	1.3, 0.9–1.9
Zahm 1993 [18] ^1^	4 cases/12 controls	1.2, 0.3–4.4
*Chloramben*		
Cantor 1992 [27] ^3^	Handled prior to 1965: 16 cases/19 controls	2.0, 1.0–4.0
De Roos 2003 [30]	34 cases/81 controls	0.9, 0.5–1.6
*Dicamba*		
Cantor 1992 [27] ^3^	Handled prior to 1965: 7 cases/7 controls	2.8, 1.0–8.1
McDuffie 2001 [43]	26 cases/50 controls	1.6, 1.0–2.6
De Roos 2003 [30]	39 cases/79 controls	1.2, 0.6–2.3
**Dinitroaniline herbicides**		
*Dinitroanilines, group*		
Cantor 1992 [27]	46 cases/88 controls	1.2, 0.8–1.8
McDuffie 2001 [43]	11 cases/31 controls	1.2, 0.6–2.4
*Trifluralin*		
Cantor 1992 [27] ^3^	Handled prior to 1965: 14 cases/23 controls	1.5, 0.8–3.1
Zahm 1993 [18] ^1^	3 cases/24 controls	0.5, 0.1–1.7
McDuffie 2001 [43]	11 cases/31 controls	1.1, 0.5–2.2
De Roos 2003 [30]	52 cases/120 controls	0.9, 0.5–1.6
Mills 2005 [43,45]	NR	0.9, 0.4–1.8
**Triazine herbicides**		
*Triazine herbicides, group*		
Hoar 1986 [34]	14 cases/43 controls	2.5, 1.2–5.4
Cantor 1992 [27]	64 cases/133 controls	1.1, 0.8–1.6
Zahm 1993 [18] **^1^**	12 cases/38 controls	1.2, 0.6–2.6
Orsi 2009 [46]	17 cases /20 controls	1.9, 0.9–3.8
*Atrazine*		
Zahm 1993 [59]	130 cases/249 controls	1.4, 1.1–1.8
*Cyanazine*		
De Roos 2003 [30]	37 cases/96 controls	0.6, 0.3–1.0
*Metribuzin*		
De Roos 2003 [30]	20 cases/53 controls	0.8, 0.4–1.7
*Simazine*		
Mills 2005 [45]	NR	1.7, 0.9–3.0
**Urea herbicides**		
*Urea herbicides*		
Cantor 1992 [27]	5 cases/18 controls	0.6, 0.2–1.6
Orsi 2009 [46]	5 cases/7 controls	1.8, 0.5–6.0
*Linuron*		
De Roos 2003 [30]	5 cases/22 controls	0.3, 0.1–1.2
**Other herbicides**		
*Bentazon*		
Cantor 1992 [27]	22 cases/58 controls	0.7, 0.3–1.5
*Nitrofen*		
Mills 2005 [45]	NR	1.2, 0.6–2.5
*Paraquat*		
De Roos 2003 [30]	2 cases/15 controls	0.1, 0.2–0.7
*Quaternary ammonium compounds, group*		
Orsi 2009 [46]	4 cases/12 controls	0.7, 0.2–2.3
*Sodium chlorate*		
De Roos 2003 [30]	8 cases/7 controls	4.1, 1.3–13.6
*Uracil herbicides*		
Hoar 1986 [34]	19 cases/114 controls	1.3, 0.7–2.5
**FUNGICIDES**		
**Aldehyde fungicides**		
*Aldehyde fungicides, group*		
McDuffie 2001 [43]	7 cases/25 controls	0.9, 0.4–2.3
*Formaldehyde*		
McDuffie 2001 [43]	7 cases/25 controls	0.9, 0.4–2.3
**Amide fungicides**		
*Amide fungicides, group*		
McDuffie 2001 [43]	30 cases/58 controls	1.7, 1.0–2.8
*Captan*		
McDuffie 2001 [43]	20 cases/24 controls	2.5, 1.3–4.8
Mills 2005 [45]	NR	0.9, 0.5–1.6
*Vitavax*		
McDuffie 2001 [43]	10 cases/39 controls	0.8, 0.4–1.9
**Carbamate and dithiocarbamate fungicides**		
*Carbamate fungicides*		
Orsi 2009 [46]	15 cases/17 controls	1.8, 0.9–3.7
*Maneb*		
Mills 2005 [45]	NR	1.1, 0.6–2.1
*Mancozeb*		
Mills 2005 [45]	NR	0.9, 0.5–1.9
**Triazole fungicides**		
*Triazole fungicides, group*		
Orsi 2009 [46]	8 cases/9 controls	1.9, 0.7–5.3
*Mecoprop*		
Pahwa 2012 [47]	51 cases/81 controls	2.3, 1.5–3.3
**Mercury containing fungicides**		
*Mercury fungicides, group*		
McDuffie 2001 [43]	18 cases/48 controls	1.3, 0.7–2.3
*Mercury dust*		
McDuffie 2001 [43]	15 cases/39 controls	1.2, 0.6–2.4
*Mercury liquid*		
McDuffie 2001 [43]	8 cases/22 controls	1.4, 0.7–3.2
**Fumigant fungicides**		
*Methyl bromide*		
Mills 2005 [45]	NR	1.5, 0.8–2.7
*Dichloro-propane*		
Mills 2005 [45]	NR	0.9, 0.5–1.7
**Other fungicides**		
*Chlorothalonil*		
Mills 2005 [45]	NR	1.2, 0.6–2.2
*Sulfur compounds*		
McDuffie 2001 [43]	17 cases/21 controls	2.8, 1.4–5.6
**INSECTICIDES**		
**Arsenicals**		
*Acetoarcenate*		
De Roos 2003 [30]	41 cases/68 controls	1.4, 0.9–2.3
*Arsenic*		
Hardell 2002 [33]	8 cases/10 controls	1.8, 0.7–4.5
Eriksson 2008 [32]	7 cases/5 controls	1.6, 0.5–5.2
*Lead arsenate*		
De Roos 2003 [30]	9 cases/25 controls	0.5, 0.2–1.2
**Botanical insecticides**		
*Nicotine*		
Cantor 1992 [27]	31 cases/47 controls	1.5, 0.9–2.5
Cantor 1992 [27] ^3^	Handled prior to 1965: 28 cases/36 controls	1.8, 1.0–3.0
*Pyrethrine*		
De Roos 2003 [30]	6 cases/12 controls	1.0, 0.3–3.2
Eriksson 2008 [32]	15 cases/10 controls	1.7, 0.8–3.9
*Rotenone*		
Cantor 1992 [27]	12 cases/23 controls	0.5, 2.2–1.0
**Carbamate insecticides**		
*Carbamate insecticides, group*		
McDuffie 2001 [43]	37 cases/60 controls	1.9, 1.2–3.0
Zahm 1993 [18] ^1^	7 cases/17 controls	1.6, 0.6–4.4
Zheng 2001 [60]	89 cases/172 controls	1.6, 1.0–2.4
*Bufencarb*		
De Roos 2003 [30]	6 cases/12 controls	1.1, 0.3–3.7
*Carbaryl*		
Cantor 1992 [27] ^3^	Handled prior to 1965: 7 cases/4 controls	3.8, 1.1–13.6
De Roos 2003 [30]	30 cases/57 controls	1.0, 0.5–1.9
McDuffie 2001 [43]	25 cases/34 controls	2.1, 1.2–3.7
*Carbofuran*		
Cantor 1992 [27] ^3^	Handled prior to 1965: 28 cases/63 controls	1.0, 0.6–1.7
McDuffie 2001 [43]	9 cases/18 controls	1.6, 0.7–3.9
Zheng 2001 [60]	66 cases/131 controls	1.6, 1.1–2.3
*Methomyl*		
McDuffie 2001 [43]	37 cases/60 controls	2.1, 1.2–3.7
**Fly spray**		
Cantor 1992 [27]	185 cases/394 controls	1.1, 0.9–1.4
Cantor 1992 [27] ^3^	Handled prior to 1965: 173 cases/368 controls	1.1, 0.9–1.4
**Organochlorine insecticides**		
*Organochlorine insecticides, group*		
Cantor 1992 [27]	150 cases/162 controls	1.3, 1.0–1.7
Zahm 1993 [18] ^1^	20 cases/46 controls	1.6, 0.8–3.1
Orsi 2009 [46]	15 cases/17 controls	1.8, 0.9–3.8
Purdue 2007 [51]	58 cases/44 non cases	0.8, 0.5–1.3
Pahwa 2012 [47]	106 cases/276 controls	1.3, 1.0–1.7
*Aldrin*		
Cantor 1992 [27] ^3^	Handled prior to 1965: 34 cases/59 controls	1.3, 0.8–2.1
McDuffie 2001 [43]	10 cases/6 controls	4.2, 1.5–12.0
De Roos 2003 [30]	47 cases/97 controls	1.1, 0.7–1.7
Purdue 2007 [51]	21 cases/79 non-cases	0.6, 0.3–1.0
*Chlordane*		
Woods 1987 [57]	NR	1.6, 0.7–3.8
Cantor 1992 [27] ^3^	Handled prior to 1965: 22 cases/22 controls	2.2, 1.2–4.2
McDuffie 2001 [43]	36 cases/105 controls	1.1, 0.7–1.7
De Roos 2003 [30]	21 cases/26 controls	1.7, 0.9–3.2
Purdue 2007 [51]	27 cases/73 non-cases	0.7, 0.4–1.2
*DDT*		
Woods 1987 [57]	Not reported	1.8, 1.0–3.2
Cantor 1992 [27] ^3^	Handled prior to 1965: 68 cases/123 controls	1.3, 0.9–1.8
Persson 1993 [50]	4 case/3 controls	2.0, 0.2–18.9
Baris 1998 [20]	161 cases/340 controls	1.2, 1.0–1.6
Hardell 2002 [33]	77 cases/138controls	1.2, 0.9–1.7
De Roos 2003 [30]	98 cases/226 controls	1.0, 0.7–1.3
Purdue 2007 [51]	37 cases/63 noncases	0.9, 0.6–1.5
Eriksson 2008 [32]	50 cases/37 controls	1.5, 0.9–2.3
Pahwa 2012 [47]	33 cases/59 controls	1.7, 1.1–2.7
*Dieldrin*		
Cantor 1992 [27] ^3^	Handled prior to 1965: 10 cases/13 controls	1.9, 0.8–4.4
De Roos 2003 [30]	21 cases/39 controls	1.8, 0.8–3.9
Purdue 2007 [51]	7 cases/92 controls	0.6, 0.2–1.3
*Heptachlor*		
Cantor 1992 [27] ^3^	Handled prior to 1965: 14 cases/25 controls	1.3, 0.6–2.6
De Roos 2003 [30]	25 cases/43 controls	1.3, 0.7–2.2
Purdue 2007 [51] ^2^	18 cases/82 noncases	0.8, 0.4–1.4
*Lindane*		
Cantor 1992 [27] ^3^	Handled prior to 1965: 14 cases/25 controls	2.2, 1.0–4.7
Blair 1998 [23]	93 cases/151 controls	1.5, 1.1–2.0
McDuffie 2001 [43]	15 cases/23 controls	2.1, 1.0–4.2
Rafnsson 2006 [52]	37 cases/133 controls	3.5, 1.4–9.0
Purdue 2007 [51]^ 2^	24 cases/76 controls	1.3, 0.8–2.1
*Methoxychlor*		
McDuffie 2001 [43]	65 cases/201 controls	1.0, 0.7–1.4
De Roos 2003 [30]	9 cases/16 controls	1.2, 0.5–2.7
*Toxaphene*		
Cantor 1992 [27] ^3^	Handled prior to 1965: 6 cases/5 controls	2.4, 0.7–8.2
De Roos 2003 [30]	10 cases/13 controls	1.5, 0.6–3.5
Purdue 2007 [51]^ 2^	24 cases/75 controls	1.5, 0.9–2.5
**Organophosphorus insecticides**		
Organophosphorus insecticides		
Zahm 1993 [18] ^1^	14 cases/43 controls	1.2, 0.6–2.5
Waddell 2001 [56]	158 cases/279 controls	l.5, 1.2–1.9
Orsi 2009 [46]	20 cases/24 controls	1.7, 0.9–3.3
Pahwa 2012 [47]	92 cases/169 controls	1.9, 1.4–2.6
*Chlorpyrifos*		
Waddell 2001 [56]	7 cases/8 controls	3.2, 1.1–9.2
Lee 2004 [38] ^2^	37 participants	1.0, 0.6–1.7
*Coumaphos*		
Cantor 1992 [27] ^3^	Handled prior to 1965: 3 cases/5 controls	1.5, 0.3–6.3
Waddell 2001 [56]	23 cases/37 controls	1.7, 1.0–2.9
*Crufomate*		
Waddell 2001 [56]	5 cases/8 controls	1.6, 0.5–4.9
*Diazinon*		
Cantor 1992 [27] ^3^	Handled prior to 1965: 14 cases/12 controls	2.6, 1.2–5.9
McDuffie 2001 [43]	18 cases/28 controls	1.7, 0.9–3.2
Waddell 2001 [56]	60 cases/93 controls	1.7, 1.2–2.5
Mills 2005 [45]	NR	1.4, 0.8–2.5
*Dichlorvos*		
Cantor 1992 [27] ^3^	Handled prior to 1965: 12 cases/17 controls	1.8, 0.8–3.9
Waddell 2001 [56]	23 cases/51 controls	1.0, 0.6–1.7
Koutros 2008 [37] ^2^	6 exposed cases	NR
*Dimethoate*		
McDuffie 2001 [43]	22 cases/50 controls	1.2, 0.7–2.1
Waddell 2001 [56]	12 cases/22 controls	1.8, 0.9–3.8
*Disulfoton*		
Waddell 2001 [56]	7 cases/13 controls	2.0, 0.8–5.3
*Ethoprop*		
Waddell 2001 [56]	7 cases/17 controls	0.9, 0.4–2.3
*Famphur*		
Waddell 2001[56]	18 cases/47 controls	1.0, 0.5–1.8
*Fensulfothion*		
Waddell 2001 [56]	4 cases/4 controls	2.0, 0.5–8.2
*Fonofos*		
Waddell 2001 [56]	43 cases/67 controls	1.7, 1.1–2.6
*Malathion*		
Cantor 1992 [27] ^3^	Handled prior to 1965: 11 cases/9 controls	2.9, 1.1–7.4
Waddell 2001 [56]	91 cases/147 controls	1.6, 1.2–2.2
Mills 2005 [45]	NR	1.8, 1.0–3.2
Pahwa 2012 [47]	72 cases/127 controls	2.0, 1.4–2.7
*Methyl parathion*		
Mills 2005 [45]	NR	0.6, 0.3–1.2
*Parathion*		
Waddell 2001 [56]	5 cases/8 controls	2.9, 0.9–9.7
*Phorate*		
Cantor 1992 [27] ^3^	Handled prior to 1965: 9 cases/12 controls	1.8, 0.7–4.5
Waddell 2001 [56]	44 cases/97 controls	1.1, 0.8–1.7
*Ronnel*		
Waddell 2001 [56]	6 cases/11 controls	1.3, 0.5–3.6
*Terbufos*		
Waddell 2001 [56]	32 cases/97 controls	1.1, 0.7–1.8
*Tetrachlorvinphos*		
Waddell 2001 [56]	9 cases/17 controls	1.8, 0.7–4.7
*Toxaphene*		
Mills 2005 [45]	NR	0.9, 0.5–1.9
*Trichlorfon*		
Cantor 1992 [27] ^3^	Handled prior to 1965: 6 cases/5 controls	2.4, 0.7–8.2
Waddell 2001 [56]	7 cases/11 controls	1.8, 0.7–4.7

Notes: 2,4-D, 2,4-Dichlorophenoxyacetic acid; 2,4,5-T, 2,4,5-Trichlorophenoxyacetic acid; EPTC, S-Ethyl dipropylthiocarbamate; MCPA, 2-methyl-4-chlorophenoxyacetic acid; DDT, dichlorodiphenyltrichloroethane; NHL, non-Hodgkin lymphoma; NR, Not reported; **^1^** Only women included in analysis; **^2^** Cohort study; **^3^** Effect estimate not included in the meta-analysis; another estimate from the same paper with a larger number of exposed cases was used.

### 3.4. Meta Analyses

When there was more than one effect estimate for a chemical group or active ingredient, the estimates shown in Table 3 and Table 4 were combined to produce meta-analytic summary estimates and 95% CIs (Table 5). 

The strongest meta RR estimates were associated with subtypes of NHL. There was a positive association between exposure to organophosphorus herbicide, glyphosate, and B cell lymphoma (2.0, 95% CI: 1.1–3.6, CLR: 3.2). Phenoxy herbicide exposures were associated with B cell lymphoma (1.8, 95% CI: 1.2–2.8, CLR: 2.4), lymphocytic lymphoma (1.8, 95% CI: 0.9–3.5, CLR: 3.8), and diffuse large B-cell lymphoma (DLBCL; 2.0, 95% CI: 1.1–3.7, CLR: 3.3). All these effect estimates were relatively precise, with CLRs < 4.

**Table 5:** Meta analytic summary estimates of association between herbicides and insecticides with non-Hodgkin lymphoma.

Chemical group or active ingredient	Meta Risk Ratio estimate, 95% CI	I^2^	Papers contributing
**HERBICIDES**			
***Amide herbicides***			
Amide herbicides	1.3, 0.8–1.9	22.2%	[18,27,34,46]
Alachlor	0.9, 0.6–1.3	43.0%	[30,39]
***Aromatic acid herbicides***			
Benzoic acid herbicides	1.3, 0.9–1.9	0.0%	[18,27,34,46]
Dicamba	1.4, 1.0–2.1	0.0%	[30,43]
***Carbamate/thiocarbamate herbicides***			
Carbamate/thiocarbamate herbicides	1.4, 1.1–2.0	0.0%	[18,43,60]
***Dinitroanilines***			
Dinitroanilines	1.2, 0.8–1.7	0.0%	[27,43]
Trifluralin	0.9, 0.6–1.3	0.0%	[18,30,43,45]
***Organophosphorus herbicides***			
Glyphosate	1.5, 1.1–2.0	32.7%	[30,31,32,33,43,46]
Glyphosate-association with B cell lymphoma	2.0, 1.1–3.6	0.0%	[32,63]
***Phenoxy herbicides***			
Phenoxy herbicides	1.4, 1.2–1.6	37.7%	[27,32,33,34,44,46,47,48,49,50,57,59]
Phenoxy herbicides, association with B cell lymphoma	1.8, 1.2–2.8	0.0%	[32,63]
Phenoxy herbicides, association with DLBCL	2.0, 1.1–3.7	0.0%	[32,63]
Phenoxy herbicides, association with lymphocytic lymphoma	1.8, 0.9–3.5	0.0%	[32,63]
2,4-D	1.4, 1.0–1.9	61.5%	[27,44,45,47,58]
MCPA	1.5, 0.9–2.5	54.4%	[30,32,33,44,47]
***Triazine herbicides***			
Triazine herbicides	1.5, 1.0, 2.1	38.5%	[18,27,34,46]
**Urea herbicides**			
Urea herbicides, group	1.0, 0.3–2.9	43.4%	[27,46]
**INSECTICIDES**			
***Arsenicals***			
Arsenic	1.7, 0.8–3.6	0.0%	[32,33]
***Botanical insecticides***			
Pyrethrine	1.4, 0.8–2.8	0.0%	[30,32]
***Carbamate insecticides***			
Carbamate insecticides, group	1.7, 1.3–2.3	0.0%	[18,43,60]
Carbaryl	1.7, 1.3–2.3	0.0%	[43,60]
Carbofuran	1.6, 1.2–2.3	0.0%	[43,60]
***Organophosphorus insecticides***			
Organophosphorus insecticides, group	1.6, 1.4–1.9	0.0%	[18,46,47,56]
Chlorpyrifos	1.6, 0.6–4.9	72.0%	[38,56]
Diazinon	1.6, 1.2–2.2	0.0%	[43,45,56]
Dimethoate	1.4, 0.9–2.1	0.0%	[43,56]
Malathion	1.8, 1.4–2.2	0.0%	[45,47,56]
***Organochlorine insecticides***			
Organochlorine insecticides, group	1.3, 1.0–1.5	19.6%	[18,27,46,47,51]
DDT	1.3, 1.1–1.5	0.0%	[20,32,33,47,50,51,57]
DDT-association with B cell lymphoma	1.4, 1.0–2.0	0.0%	[20,32,63]
DDT-association with DLBCL	1.2, 0.9–1.7	0.0%	[20,32]
DDT-association with follicular lymphoma	1.5, 1.0–2.4	26.6%	[20,32]
Methoxychlor	1.0, 0.7–1.4	0.0%	[30,43]
Aldrin	1.0, 0.4–2.7	84.6%	[30,43,51]
Chlordane	1.1, 0.8–1.6	32.5%	[30,43,51,57]
Dieldrin	1.1, 0.4–3.1	67.6%	[30,51]
Heptachlor	0.9, 0.6–1.5	0.0%	[30,51]
Lindane	1.6, 1.2–2.2	26.0%	[23,43,51,52]
Toxaphene	1.4, 0.9–2.1	0.0%	[30,45,51]
***Amide fungicides***			
Captan	1.5, 0.5–4.2	82.5%	[43,45]

Notes: 2,4-D, 2,4-Dichlorophenoxyacetic acid; 2,4,5-T, 2,4,5-Trichlorophenoxyacetic acid; DDT, dichlorodiphenyltrichloroethane; EPTC, s-ethyl dipropylthiocarbamate; MCPA, 2-methyl-4-chlorophenoxyacetic acid; NHL, non-Hodgkin lymphoma; DLBCL, diffuse large B cell lymphoma; OC, Organochlorine; OP; Organophosphorus.

The meta RR estimates (95% CI) of association between phenoxy herbicide exposure and NHL subtypes were more positive than those for NHL overall, although the estimate of association with NHL overall was more precise (meta RR, 95% CI: 1.4, 1.2–1.6, CLR: 1.4). Only two papers contributed to each of the estimates of association between phenoxy herbicide exposures and NHL subtypes, and 12 papers contributed to the meta RR estimates for the relationship between phenoxy herbicide exposure and NHL overall. 

There was a positive and relatively precise association between NHL and the phenoxy herbicide 2-methyl-4-chlorophenoxyacetic acid (MCPA) (meta RR, 95% CI: 1.5, 0.9–2.5, CLR: 2.6). Five estimates contributed to this summary estimate; an I^2^ value of 54.4% indicates some inconsistency in the effect estimates. The forest plot for the meta-analysis of MCPA, along with plots for meta-analyses of phenoxy herbicicides as a group, the phenoxy herbicide 2,4-D, glyphosate, organochlorine insecticides as a group, and the organochlorine insecticide DDT, are presented in Supplementary Figure S1. 

In addition to assessing the association of ever exposure to MCPA with NHL, Hardell *et al.* [33] investigated dose-response relationships between number of days of exposure; they observed increasing odds in association with increased number of days of MCPA exposure (Table 2). In similar analyses, Eriksson *et al.* [32] and Mcduffie *et al.* [43] did not observe dose-response relationship between days/year of MCPA exposure and NHL. 

There was a positive but less precise estimate of association between arsenic and NHL (meta RR, 95% CI: 1.7, 0.8–3.6, CLR: 4.4). Meta estimates of association between NHL and carbamate insecticides and carbaryl, a carbamate insecticide, were nearly identical (meta RR, 95% CI: 1.7, 1.3–2.3, CLR: 1.8) and both were positive and precise. Estimates from three papers contributed to the meta analysis of carbamate insecticides. The I^2^ value was 0%, indicating consistency in effect estimates. Carbofuran, another carabamate insecticide, was positively associated with NHL (meta RR, 95% CI: 1.6, 1.2–2.3, CLR: 2.0). However, in two investigations from the Agricultural Health Study that reported estimates of association with tertiles of lifetime days of exposure to carbofuran [25] and carbaryl [42], the relationships were imprecise and there was a lack of a dose-response relationship (Table 2).

There were positive and precise estimates of association between NHL and organophosphorus insecticides (meta RR, 95% CI: 1.6, 1.4–1.9, CLR: 1.4), and the organophosphorus insecticides diazinon (meta RR, 95% CI: 1.6, 1.2–2.2, CLR: 1.8), and malathion (meta RR, 95% CI: 1.8, 1.4–2.2, CLR: 1.5). Although Fritschi *et al.* [62] studied the relationship between organophosphorus insecticides and NHL, we did not include the estimate from their paper in the meta analysis because they investigated the association with exposure in three categories (no exposure, non-substantial exposure, substantial exposure). Fritschi *et al.* [62] reported a positive but imprecise estimate for substantial exposure *versus* no exposure (odds ratio, 95% CI: 2.1, 0.8–5.7, CLR: 7.3). The meta RR estimate of association between NHL and the organophosphorus insecticide chlorpyrifos was positive but imprecise (meta RR, 95% CI: 1.6, 0.6–4.9, CLR: 8.9). There was a positive and precise association with lindane, an organochlorine insecticide (meta RR, 95% CI: 1.6, 1.2–2.2, CLR: 1.8); estimates of association with other organochlorine insecticides were closer to the null. 

### 3.5. Sensitivity Analyses

We conducted sensitivity analyses to examine the effect of gender (Supplementary Table S1), study design (Supplementary Table S2), diagnosis period (Supplementary Table S3), geographic region (Supplementary Table S4), source for controls in case-control studies (Supplementary Table S5) and/or the effect of using alternative papers that represent the same study population (Supplementary Table S6). For the most part, meta-estimates were robust. 

#### 3.5.1. Gender

When we subset the analyses of associations between NHL and amide herbicides to the two studies that included men only, the association became more positive but less precise (meta RR, 95% CI: moved from 1.3, 0.8–1.9, CLR: 2.3 to 1.7, 0.7–3.8, CLR: 5.3). Restricting to all male studies moved the summary estimate of the relationship with aldrin up and across the null; however, the estimate in the sensitivity analysis was too unstable to interpret (meta RR, 95% CI: moved from 1.0, 0.4–2.7, CLR: 7.8 to 1.4, 0.2–11.1, CLR: 65.0). Restricting the analysis to studies that included men and women caused the meta RR estimate of association between NHL and 2,4-D to become more positive but less precise; it moved from 1.4, 1.0–1.9, CLR: 1.9 to 1.8, 0.5–7.5, CLR: 16.7. We were not able to conduct sensitivity analyses for female only studies, since only one paper reported results for women only [18]. 

#### 3.5.2. Study Design

Nearly all of the studies that contribute to the meta estimates were case control in design. The only cohort study was the Agricultural Health Study. In nearly all of the analyses of data from the Agricultural Health Study, exposure was defined using multiple categories. However, in the papers on glyphosate [31], chlorpyrifos [38], organochlorine insecticides, aldrin, chlordane, dieldrin, lindane, and toxaphene [51], the association with ever/never use of exposure was analyzed. For the most part, restricting analyses to case control studies did not cause the meta estimate to change substantially (Supplementary Table S2). However, the magnitude of the meta RR for aldrin moved up and away from the null, but became more imprecise (it moved from 1.0, 0.4–2.7, CLR: 6.8 to 1.4, 0.2–11.1, CLR: 55.5). For lindane it changed from 1.6, 1.2–2.2, CLR: 1.8 to 1.9, 1.2–2.9, CLR: 2.4. 

#### 3.5.3. Diagnosis Period

We also investigated the sensitivity of the meta-analytic estimates to decade of cancer diagnosis (Supplementary Table S3). For the most part, estimates were robust. However, when we subset the meta-analysis of glyphosate to the two papers in which cases were diagnosed from 1975–1989, the meta RR, 95% CI changed from 1.5, 1.1–2.0, CLR: 1.8 to 2.3, 1.4–4.0, CLR: 3.0. Similarly, for the phenoxy herbicide 2,4-D, when we included estimates from the three papers with diagnosis periods from 1975 to 1989, the summary estimate was more positive but less precise (meta RR, 95% CI: 1.8, 1.0–3.1, CLR: 3.2) compared to the full meta-analysis estimate (1.4, 95% CI: 1.0–1.9; CLR: 1.9). 

#### 3.5.4. Geographic Area

We investigated the impact of geographic area on the meta-analytic RR estimates (Supplementary Table S4). For glyphosate exposure, including estimates from papers that reported results from Swedish studies caused the estimate to become more positive; it moved from 1.5, 95% CI: 1.1–2.0, CLR: 1.8 to 2.2, 95% CI: 1.3–3.8, CLR: 2.9. Similarly, restricting estimates of the relationship between NHL and phenoxy herbicide exposure to Sweden caused the estimate to become more positive; it changed from 1.4, 95% CI: 1.2–1.6, CLR: 1.4 to 1.9, 1.4–2.4, CLR: 1.7. When we restricted estimates of association with MCPA to those that came from North American studies, the meta RR moved towards the null, from 1.5, 0.9–2.5, CLR: 2.6 to 1.1, 0.7–1.8, CLR: 2.7. In contrast, restricting to European and Swedish studies caused the estimate of association with MCPA to become more positive (meta RR, 95% CI: 1.9, 0.9–3.8, CLR: 4.1 and 2.7, 1.6–4.4, CLR: 2.7 respectively). When we included estimates of association with aldrin that came from studies conducted in the USA, the estimate became more precise but moved down and away from the null (meta RR, 95% CI: 1.0, 95% CI: 0.4–2.7, CLR: 7.8 changed to 0.5, 95% CI: 0.4–0.8, CLR: 2.3). 

#### 3.5.5. Source of Controls in Case Control Studies

Only two papers reported results from case control studies in which controls were selected from the hospital [46,48]. The meta-analytic RR estimates remained robust when we restricted the estimates to those resulting from population-based case-control studies (Supplementary Table S5).

#### 3.5.6. Alternative Papers

In several cases, analyses of the same study populations were represented in multiple papers. For the meta-analyses, we included the result(s) that represented the largest number of participants. In some cases, we selected the result from a pooled analysis instead of the individual, original studies. In other cases, use of effect estimates from the individual studies was preferable because it represented more people. We performed sensitivity analyses to evaluate the impact of replacing results from pooled analyses of multiple studies [23,30,59,60] with the original ones [27,34,58], or the original ones with the pooled analyses (Supplementary Table S6). 

When we replaced the estimate of a relationship between carbofuran exposure and NHL reported in Zheng *et al.* [60] by that reported in Cantor *et al.* [27] the relationship became weaker and less precise; the meta RR and 95% CI changed from 1.6, 1.2–2.3, CLR: 2.0 to 1.1, 0.7–1.8, CLR: 2.4. Using the estimate reported in De Roos *et al.* [30] yielded a similar result (meta RR, 95% CI changed to 1.1, 0.6–2.0, CLR: 3.1). For the relationship between aldrin and NHL, we replaced the estimate reported in De Roos *et al.* [30] by that reported by Cantor *et al.* [27]; the estimate moved from a null relationship to a positive one (meta RR, 95% CI changed from 1.0, 0.4–2.7 to 1.3, 0.5–2.9). 

## 4. Discussion

This systematic review and series of meta-analyses show that there is consistent evidence of positive associations between NHL and carbamate insecticides, organophosphorus insecticides, lindane, an organochlorine insecticide, and MCPA, a phenoxy herbicide. Our results represent an important contribution to a growing body of literature on agricultural exposures associated with cancer. Past review papers and meta-analyses have identified positive associations between NHL and farming related exposures, including fertilizers, chemicals, and animals [5], and occupational exposures to pesticides [6]. 

We extracted estimates of association of NHL with individual pesticide chemical groups or active ingredients from 44 papers that reported analyses of results from 17 independent studies. The studies represented data collected in 12 countries, the majority of which were located in either Europe or North America. Several of the papers that we identified were related to one another; many used data from the same cohort study, the Agricultural Health Study, and several others pooled the same data from individual studies. Thus, although this review identified 44 papers, it also highlights the need for additional epidemiologic studies in a larger variety of geographic locations. 

In the papers from which we extracted information, estimates of associations with NHL were reported with 13 herbicide chemical groups and 28 herbicide active ingredients, five fungicide groups and 12 fungicide active ingredients, and three insecticide groups and 40 insecticide active ingredients. More than 1,700 active ingredients are listed in Alan Wood’s compendium of pesticide common names, although not all of these are necessarily used in agriculture or currently registered for use in any or all countries [13]. Many chemicals remain for consideration in future epidemiologic analyses of associations between NHL and pesticides. It would be useful to identify pesticides to investigate by ranking, by country, the most commonly used chemicals. 

The positive and precise estimate of associations of NHL with carbamate insecticides, organophosphorus insecticides, and lindane were robust to sensitivity analyses of gender, geographic area, and cancer diagnosis period. The positive association between MCPA and NHL was robust to a sensitivity analysis of diagnosis period, but when we restricted the meta-analysis to estimates from studies conducted in North America, the estimate moved to the null. 

Consistent with the results from the meta-analysis of lindane exposure, analyses of data from the American cohort, the Agricultural Health Study, revealed a positive dose-response relationship between NHL and intensity weighted lifetime days of lindane exposure, where the referent group consisted of applicators never exposed to pesticide products containing the active ingredient [51]. In this same paper, however, the estimate of association with dichotomously coded exposure to lindane was close to the null and imprecise. This difference in results within the Agricultural Health Study suggests that dichotomous classification of exposure might be too crude; the binary categories could lead to exposure misclassification and attenuated effect estimates. Because of variability in definitions and cut-points across papers, we were unable to conduct formal meta-analyses of exposures classified using multiple categories. When they were available, we reviewed estimates of dose-response relationships from the individual papers. We found that, in most of the papers in which dose-response relationships were investigated, effect estimates were imprecise due to small numbers of exposed cases within categories. 

There were positive meta RR estimates of association of NHL with two carbamate insecticides, carbaryl and carbofuran, and the organophosphorus insecticide active ingredients diazinon and malathion. However, results from analyses of Agricultural Health Study data, which were not included in the meta-analyses, did not show dose response relationships between NHL and lifetime days of exposure to carbofuran [25], carbaryl [42], diazinon [29] or malathion [21,26]. 

Some discrepancies in findings from the Agricultural Health Study compared to the other studies could be due to differences in design (cohort *versus* case-control). Differences could also be the result of different referent category compositions. All participants of the Agricultural Health Study were pesticide applicators; therefore, the referent group generally consisted of applicators who were not exposed to the pesticide active ingredient of interest. In contrast, in the papers contributing to the meta-RR estimate for carbaryl [30,43] and carbofuran [43,60], the referent groups consisted of farmers and non-farmers [30,43], or only of non-farmers [60]. In the papers contributing to the meta-analyses of malathion and diazinon, the referent categories consisted of non-farmers [56], farmers and non-farmers [43,47], and only farm-workers [45]. It is possible that, in studies that included non-farmers in the referent group, confounding by other agricultural exposures, not adjusted for in analysis, caused estimates of association to be higher than results from Agricultural Health Study analyses.

Only a handful of papers reported associations of pesticides with NHL subtypes; this is probably due to small sample sizes. Our meta-analyses of these relationships suggested the need for further studies of this kind, especially since some of the strongest relationships were seen with the most common subtype of NHL, B cell lymphoma and, more specifically, with DLBCL. NHL are a heterogeneous group of malignancies that include multiple subtypes with varied characteristics and possibly diverse etiologies [4]. Consequently, the overall group of neoplasms represented by NHL might be too diverse as a study endpoint to adequately detect associations with pesticide exposures in epidemiologic analyses. Some but not all specific subtypes of lymphoma might be associated with pesticides, and these relationships would only be revealed by analyses of the subtypes. Pooling projects that include cases of the NHL subtypes that have been classified according to the more recent and etiologically specific definitions (B-cell, T-cell, and within these, more refined subtypes of T- and B-cell neoplasms) [65] present the opportunity to perform more sensitive epidemiologic analyses and identify important relationships that may have been undetected if the cancer outcome was defined broadly as NHL overall. Such projects are particularly attractive for studying rarer subtypes (*i.e.*, T-cell). To this end, a pooling project within the AGRICOH consortium [64] is currently underway to investigate these associations. 

There are various sources of heterogeneity across the studies that contributed to these meta-analyses; these include gender, region, cancer diagnosis period, exposure assessment methods, exposure definitions, referent groups, study populations, and/or analysis adjustment sets. Different activity patterns, which might cause differences in exposure, combined with different biological mechanisms, could result in between-gender differences in chemical exposure and disease risk associations. Pesticide use, application, and handling patterns, regulations and legislation, demographics and genetics differ by region, which could contribute to area-specific differences in associations. In the papers that contributed to the meta analyses, a variety of exposure assessment methods were used; these included self-reported chemical exposures, exposure matrices, and approximations based on number of animals raised. Differences in exposure assessment methods could influence the magnitude of effects observed, especially since some methods might be superior to others in terms of reducing the potential for exposure misclassification. Study design (case-control *versus* cohort) and source of controls in case-control studies (hospital *versus* population) could also influence the magnitude of the exposure estimates observed. In case-control studies, exposure is assessed retrospectively, which could lead to recall bias. In contrast, in the Agricultural Health Study, the only cohort included in this review, exposure was assessed when participants were cancer-free. Finally, NHL classification systems have changed over time, reflecting changes in disease definitions [1]. Recently (after year 2000), the definition of NHL has become more comprehensive. The definition now includes disease entities that were excluded from earlier definitions, such as plasma cell neoplasms (*i.e.*, multiple myeloma) and chronic lymphocytic leukemia. These malignancies are also among the most frequently reported sub-types within NHL [65]. Thus, estimates of association between pesticides and overall NHL from studies conducted in earlier periods may not be entirely comparable to estimates from research conducted since the year 2000 that used the updated NHL definition. 

We did not conduct a formal test of publication bias; it is unclear if asymmetry tests with funnel plots are useful in meta-analyses of observational studies, and it has been recommended that these tests not be used when fewer than 10 studies contribute to a meta-analysis [66]. For the most part, we believe that our review was systematic and comprehensive. 

Nevertheless, we did not identify papers that published results of studies conducted in middle- and low-income countries. It is possible that, in such regions where cancer-follow and exposure ascertainment may be particularly challenging, no studies have investigated the relationship of NHL with pesticide exposures. Restricting our literature search to articles published in English could be another reason that we did not identify studies in lower-income countries. A lack of studies in these areas is potentially alarming, since these regions are responsible for much of the world’s agricultural production [67]. Also, lympho-hematopoetic malignancies represent a substantial proportion of cancers in low- and middle-income countries. For example, based on estimates from the World Health Organization’s GLOBOCAN 2012, NHL accounted for 37.7% of the estimated prevalent cancer cases diagnosed in the past 5 years, among adults in less-developed regions (Africa, Asia excluding Japan, Latin America and the Caribbean, Melanesia, Micronesia, and Polynesia) [68]. Nevertheless, research results from higher-income countries could be transferable and have important implications for pesticide regulation and legislation world-wide, especially in low-income countries where protective equipment may be less available and/or used.

There are several mechanisms by which pesticide exposure might be associated with NHL. First, pesticides might cause chromosomal aberrations and genetic mutations. An often studied chromosomal abnormality is the t(14;18) translocation, which is particularly common among cases of follicular lymphoma and diffuse large B-cell lymphoma [69]. In a paper that used data from the Iowa/Minnesota case-control study that contributed to several of the pooled and individual analyses that we reviewed [23,30,59], Schroeder *et al.* [70] investigated the relationship between pesticide exposures and the t(14;18) translocation. Compared with controls, t(14;18) positive NHL cases but not t(14;18) negative cases had a higher odds of exposure to dieldrin, toxaphene, lindane, and atrazine. Chiu *et al.* [69,71] performed a similar analysis using data from the Nebraska-based case-control study and reported positive associations between t(14;18) positive NHL and dieldrin, toxapehen, and lindane. A second mechanism by which pesticide exposure may cause NHL is by altering cell mediated immune function. Indeed, immunological changes have been observed following short-term exposure to phenoxy herbicides (2,4-D and MCPA) among farmers [72].

The IARC Monographs have evaluated the carcinogenicity of a handful of pesticides. Of these, only arsenic and inorganic arsenic compounds have been given a Group 1 rating (carcinogenic to humans) [73]. The fumigant insecticide ethylene dibromide was classified as a group 2A carcinogen based on inadequate evidence for carcinogenicity in humans but sufficient evidence in experimental animals; the overall evaluation was upgraded to 2A (probably carcinogenic to humans) with supporting evidence from other relevant data [74]. In Volume 53 (1991) [75], the fungicide captafol was also classified as a group 2A carcinogen based on sufficient evidence in experimental animals but no available data from human studies. In this same volume, several other pesticides were classified as either group 2B (possibly carcinogenic to humans) or group 3 carcinogens (not classifiable as to its carcinogenicity)—aldicarb chlordane/heptachlor, DDT, deltamethrin, dichlorvos, fenvalerate, permethrin, thiram, ziram, atrazine, monuron, picloram, simazine, and trifluralin. The IARC monographs have classified other pesticides, including heptachlor, chlordane, and toxaphene [76], as group 2B carcinogens; in each of these cases, the 2B classification was based on inadequate evidence in humans but sufficient evidence in experimental animals. Chlorophenoxy herbicides were classified as group 2B carcinogens based on limited evidence for carcinogenicity in humans, and inadequate evidence for carcinogenicity of 2,4-D and 2,4,5-T in animals [77].Similarly, hexachlorocyclohexanes were evaluated as group 2B carcinogens due to inadequate evidence for carcinogenicity to humans, sufficient evidence for carcinogenicity to animals for the technical-grade and the alpha isomers but limited for the beta and gamma (lindane) isomers [77]. Several other pesticides, including malathion and maneb [77] have been classified as group 3 carcinogens. These evaluations took place several decades ago and there is now more epidemiologic literature that can provide information. There also remains a need for further epidemiologic research of certain chemicals, which could help to inform future evaluations. In the current systematic review, we did not observe entirely consistent trends in association for all of the active ingredients within chemical groups. Furthermore, classification of active ingredients into groups is subjective and there is not a consistent and established scheme for doing so. Therefore, evaluations of individual active ingredients rather than chemical groups might be more useful. 

### Limitations and Strengths

Because of variability in definitions and metrics that were used in published papers, we were not able to consider additional exposure definitions, such as exposure lags, duration of exposure (e.g., number of days/year exposed), or routes of exposure (e.g., application *versus* mixing of pesticides). In an effort to use similar exposure definitions from the various papers, we only included dichotomous definitions in the meta-analyses. Since dose-response relationships could not be summarized, this restricted the strength of our conclusions from an etiologic perspective. Furthermore, we were not able to conduct analyses of certain active ingredients or chemical groups due to a lack of published literature. In other cases, very few papers contributed to the meta-analyses. The largest number of papers contributing to any meta-analysis was 12 for phenoxy herbicides, followed by eight for DDT. Most meta-analyses included estimates from only two to three studies. In most papers, associations with NHL overall, rather than with subtypes of NHL, were investigated. Thus, most of our meta-analyses were of associations with NHL rather than with its subtypes, which are probably more homogeneous disease entities for assessing the relationship with pesticides. It is possible that this led to a dilution of effects, since the various NHL subtypes have diverse etiologies and some might be more strongly associated with certain pesticides than others. 

Nevertheless, this systematic review represents a novel contribution to the literature on NHL and pesticide exposure. We identified trends in the relationship of NHL and NHL subtypes with chemical groups and active ingredient groups. To our knowledge, this is the most comprehensive systematic review and meta-analysis to investigate associations with specific agricultural pesticide active ingredients. We observed fairly consistent results for certain pesticide groups and active ingredients. We evaluated the robustness of our meta-analyses by examining the sensitivity of the estimates to gender, study design, region, diagnosis period, control source in case-control studies, and paper that provided the effect estimate. 

## 5. Conclusions

We systematically reviewed more than 25 years’ worth of epidemiologic literature on the relationship between pesticide chemical groups and active ingredients with NHL. This review indicated positive associations between NHL and carbamate insecticides, OP insecticides, the phenoxy herbicide MCPA, and lindane. Few papers reported associations with subtypes of NHL; however, based on the few that did, there were strong associations between certain chemicals and B cell lymphomas. Our results show that there is consistent evidence that pesticide exposures experienced in occupational agricultural settings may be important determinants of NHL. This review also revealed clear research needs, including further investigation of some already studied pesticide active ingredients, of additional pesticides that have not yet been investigated in epidemiologic analyses, of the strength of association of pesticide exposures with subtypes of NHL, and of the relationship between NHL and pesticides in middle- and low- income areas. 

## Supplementary Files

Supplementary File 1 Supplementary Information (PDF, 341 KB)
