# Supplementary material for: Non-Hodgkin Lymphoma and Occupational Exposure to Agricultural Pesticide Chemical Groups and Active Ingredients: A Systematic Review and Meta-Analysis
Source: Int J Environ Res Public Health. 2014 Apr 23;11(4):4449–527. doi: 10.3390/ijerph110404449 (PMC4025008; doi:10.3390/ijerph110404449)
Supplement: Supplementary File 1 — Supplementary Information (PDF, 341 KB) [file ijerph-11-04449-s001.pdf]

Supplementary Information

Non–Hodgkin Lymphoma and Occupational Exposure to Agricultural Pesticide Chemical Groups and Active Ingredients: A Systematic Review and Meta–Analysis

---

S1. List of terms included in the PubMed literature search.

((((((((("agricultural workers' diseases/chemically induced"[MAJR] AND "neoplasms"[MeSH Major Topic] AND ("1980/01/01"[PDAT] : "2013/06/31"[PDAT]) AND "humans"[MeSH Terms]) OR (((("occupational exposure"[MeSH Terms] OR occupational exposure[Title/Abstract]) OR "occupational exposure"[MeSH Terms]) OR occupational exposures[Title/Abstract]) AND ("1980/01/01"[PDAT] : "2013/06/31"[PDAT]) AND "humans"[MeSH Terms])) AND (((((((("lymphoma, non–hodgkin"[MeSH Terms] AND "humans"[MeSH Terms] AND english[la]) OR (non–hodgkin[tiab] OR non–hodgkins[tiab]) AND (lymphoma[tiab] OR lymphomas[tiab])) AND ("1980/01/01"[PDAT] : "2013/06/31"[PDAT]) AND "humans"[MeSH Terms]) OR "neoplasms"[MeSH Terms]) OR neoplasm[Title/Abstract]) OR cancer morbidity[Title/Abstract]) OR cancer mortality[Title/Abstract]) AND ("1980/01/01"[PDAT] : "2013/06/31"[PDAT]) AND "humans"[MeSH Terms])) AND (((pesticid[tiab] OR pesticidal[tiab] OR pesticidal'[tiab] OR pesticidally[tiab] OR pesticidas[tiab] OR pesticide[tiab] OR pesticide/albumin[tiab] OR pesticide/animal[tiab] OR pesticide/biocide[tiab] OR pesticide/commodity[tiab] OR pesticide/crop[tiab] OR pesticide/environmental[tiab] OR pesticide/fertilizer[tiab] OR pesticide/food[tiab] OR pesticide/fruit[tiab] OR pesticide/fungicide[tiab] OR pesticide/ha[tiab] OR pesticide/heavy[tiab] OR pesticide/herbicide[tiab] OR pesticide/humic[tiab] OR pesticide/m2[tiab] OR pesticide/matrix[tiab] OR pesticide/metabolite[tiab] OR pesticide/metabolites[tiab] OR pesticide/metal[tiab] OR pesticide/mmt[tiab] OR pesticide/neurotoxin/free[tiab] OR pesticide/nitrate[tiab] OR pesticide/oxidation[tiab] OR pesticide/pathogen[tiab] OR pesticide/petroleum[tiab] OR pesticide/polymer[tiab] OR pesticide/product[tiab] OR pesticide/seed[tiab] OR pesticide/soil[tiab] OR pesticide/solvent[tiab] OR pesticide'[tiab] OR pesticide's[tiab] OR pesticideformulating[tiab] OR pesticiderelated[tiab] OR pesticides[tiab] OR pesticides/biocides[tiab] OR pesticides/chemicals[tiab] OR pesticides/commodities[tiab] OR pesticides/consumption[tiab] OR pesticides/contaminants[tiab] OR pesticides/fertilisers[tiab] OR pesticides/fertilizer[tiab] OR pesticides/fertilizers[tiab] OR pesticides/fruit[tiab] OR pesticides/fungicides[tiab] OR pesticides/herbicide[tiab] OR pesticides/herbicides[tiab] OR pesticides/insecticides[tiab] OR pesticides/metabolites[tiab] OR pesticides/metals[tiab] OR pesticides/pesticide[tiab] OR pesticides/petroleum[tiab] OR pesticides/polycyclic[tiab] OR pesticides/sample[tiab] OR pesticides/vasectomy/occupational[tiab] OR pesticides/weedicides[tiab] OR pesticides'[tiab] OR pesticidesatlas[tiab] OR pesticidestargeted[tiab] OR pesticidic[tiab] OR pesticids[tiab])

OR "pesticides"[MeSH Terms] OR pesticides[nm] OR (insecticid[tiab] OR insecticidal[tiab] OR insecticidal/acaricidal[tiab] OR insecticidal/anthelmintic[tiab] OR insecticidal/antifeedant[tiab] OR insecticidal/irritant[tiab] OR insecticidal/larvicidal[tiab] OR insecticidal/narcotic[tiab] OR insecticidal'[tiab] OR insecticidal'b[tiab] OR insecticidally[tiab] OR insecticidation[tiab] OR insecticide[tiab] OR insecticide/acaricide[tiab] OR insecticide/antifeedant[tiab] OR insecticide/ascaricide[tiab] OR insecticide/atrazine[tiab] OR insecticide/fumigant[tiab] OR insecticide/fungicide[tiab] OR insecticide/herbicide[tiab] OR insecticide/kg[tiab] OR insecticide/lipid[tiab] OR insecticide/liter[tiab] OR insecticide/miticide[tiab] OR insecticide/mosquito[tiab] OR insecticide/nematicide[tiab] OR insecticide/nematocide[tiab] OR insecticide/organophosphrus[tiab] OR insecticide/pesticide/herbicide[tiab] OR insecticide/repellant[tiab] OR insecticide/repellent[tiab] OR insecticide'[tiab] OR insecticide's[tiab] OR insecticided[tiab] OR insecticideresistance[tiab] OR insecticideresistant[tiab] OR insecticides[tiab] OR insecticides/acaricides[tiab] OR insecticides/attract[tiab] OR insecticides/larvicides[tiab] OR insecticides/mn[tiab] OR insecticides/pesticides[tiab] OR insecticides/repellents[tiab] OR insecticides'[tiab] OR insecticidetreated[tiab] OR insecticidewise[tiab] OR insecticidial[tiab] OR insecticidic[tiab] OR insecticiding[tiab] OR insecticidity[tiab] OR insecticido[tiab]) OR "insecticides"[MeSH Terms] OR insecticides[nm] OR (herbicidal[tiab] OR herbicidally[tiab] OR herbicide[tiab] OR herbicide/binding[tiab] OR herbicide/dessicant[tiab] OR herbicide/fungicide[tiab] OR herbicide/g[tiab] OR herbicide/humic[tiab] OR herbicide/insect[tiab] OR herbicide/kg[tiab] OR herbicide/micelle[tiab] OR herbicide/ml[tiab] OR herbicide/mutation[tiab] OR herbicide/nematicide[tiab] OR herbicide/outcome[tiab] OR herbicide/pesticide[tiab] OR herbicide/substrate[tiab] OR herbicide/therapeutic[tiab] OR herbicide/tio2[tiab] OR herbicide's[tiab] OR herbicided[tiab] OR herbicideh[tiab] OR herbicideh/phytocide[tiab] OR herbicideinduced[tiab] OR herbicides[tiab] OR herbicides/chlorophenols[tiab] OR herbicides/desiccants[tiab] OR herbicides/fungicides[tiab] OR herbicides/pesticides[tiab] OR herbicides'[tiab] OR herbicidetolerant[tiab] OR herbicidies[tiab] OR herbicidin[tiab] OR herbicidins[tiab] OR herbicidovorans[tiab] OR herbicids[tiab]) OR (herbicides[nm] OR herbicidins[nm]) OR (fungicid[tiab] OR fungicidal[tiab] OR fungicidal/bactericidal[tiab] OR fungicidal/fungistatic[tiab] OR fungicidal/parasiticidal[tiab] OR fungicidally[tiab] OR fungicidals[tiab] OR fungicide[tiab] OR fungicide/algicide[tiab] OR fungicide/antioxidant[tiab] OR fungicide/bactericide[tiab] OR fungicide/disinfectant[tiab] OR fungicide/oomyceticide[tiab] OR fungicide/slimicide[tiab] OR fungicide's[tiab] OR fungicideal[tiab] OR fungicideinsensitive[tiab] OR fungicides[tiab] OR fungicides/herbicides[tiab] OR fungicides'[tiab] OR fungicidial[tiab] OR fungicidic[tiab] OR fungicidicus[tiab] OR fungicidin[tiab] OR fungicidine[tiab] OR fungicidity[tiab] OR fungicido[tiab] OR fungicidy[tiab])) AND ("1980/01/01"[PDAT] : "2013/06/31"[PDAT]) AND "humans"[MeSH Terms])) NOT News[Publication Type]) NOT Congresses[Publication Type]) NOT Review[Publication Type]) AND ("1980/01/01"[PDAT] : "2013/06/31"[PDAT]) AND "humans"[MeSH Terms]) NOT "child"[MeSH Terms] AND (("1980/01/01"[PDAT] : "2013/12/31"[PDAT]) AND "humans"[MeSH Terms]))

Figure S1. Forest plots showing estimates of association between non–Hodgkin lymphoma and occupational, agricultural exposures to (**A**) phenoxy herbicides, (**B**) 2,4–D, (C) MCPA, (**D**) glyphosate, (**E**) organochlorine insecticides, and (**F**) DDT.

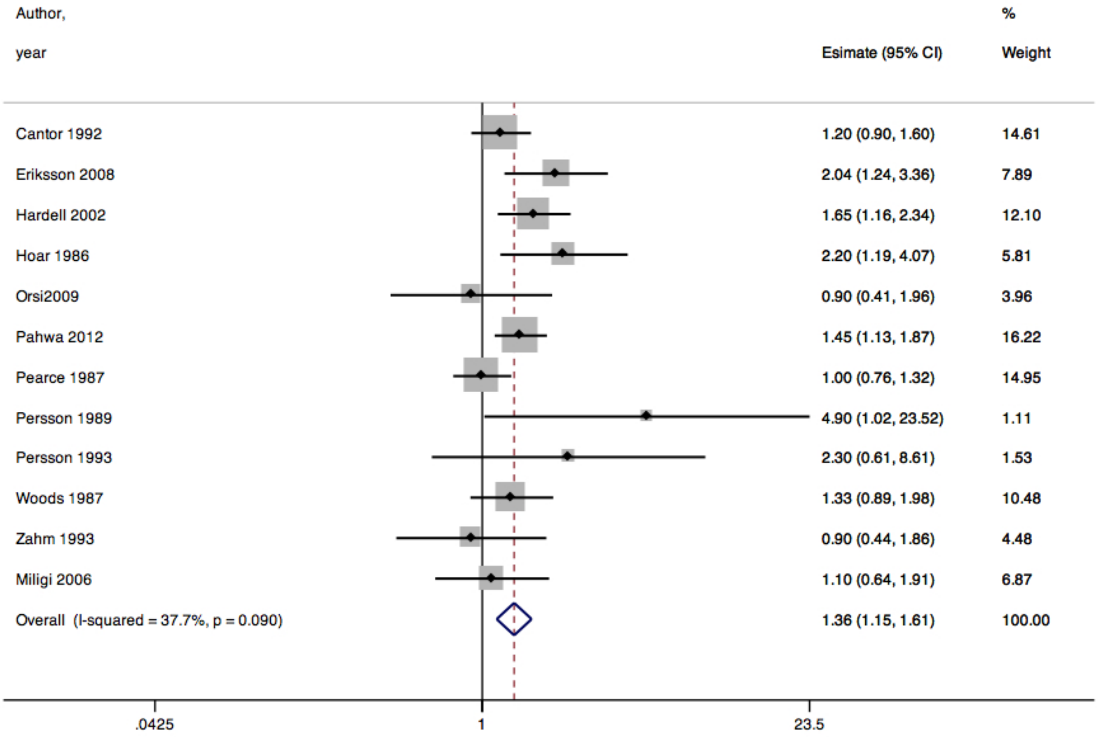

(A)

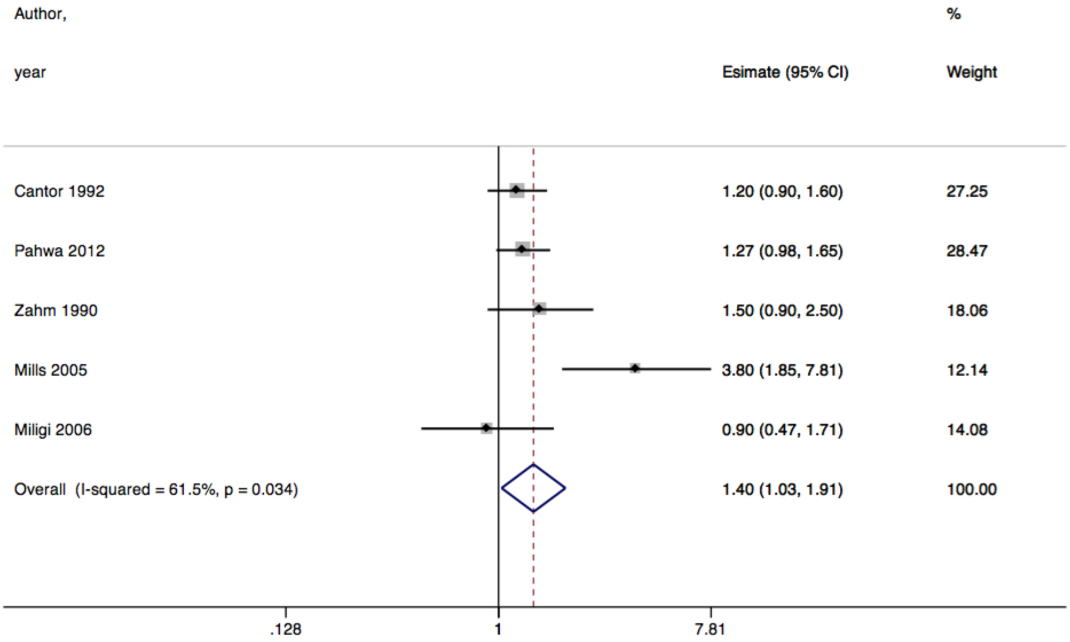

(B)

Figure S1. *Cont.*

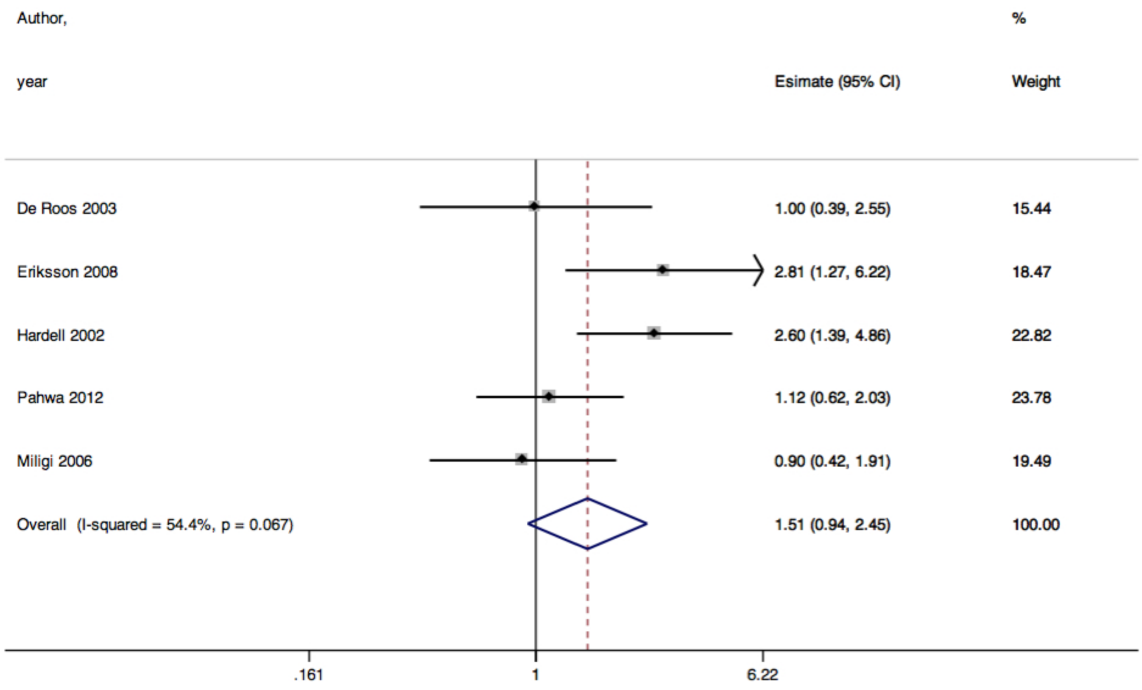

(C)

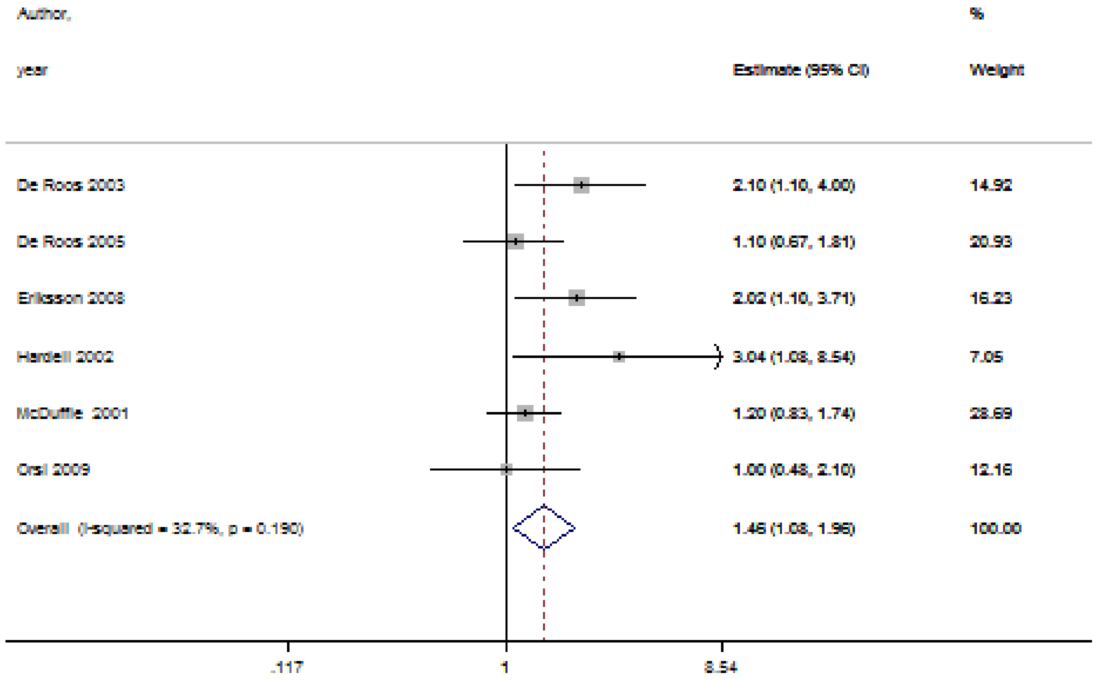

(D)

Figure S1. *Cont.*

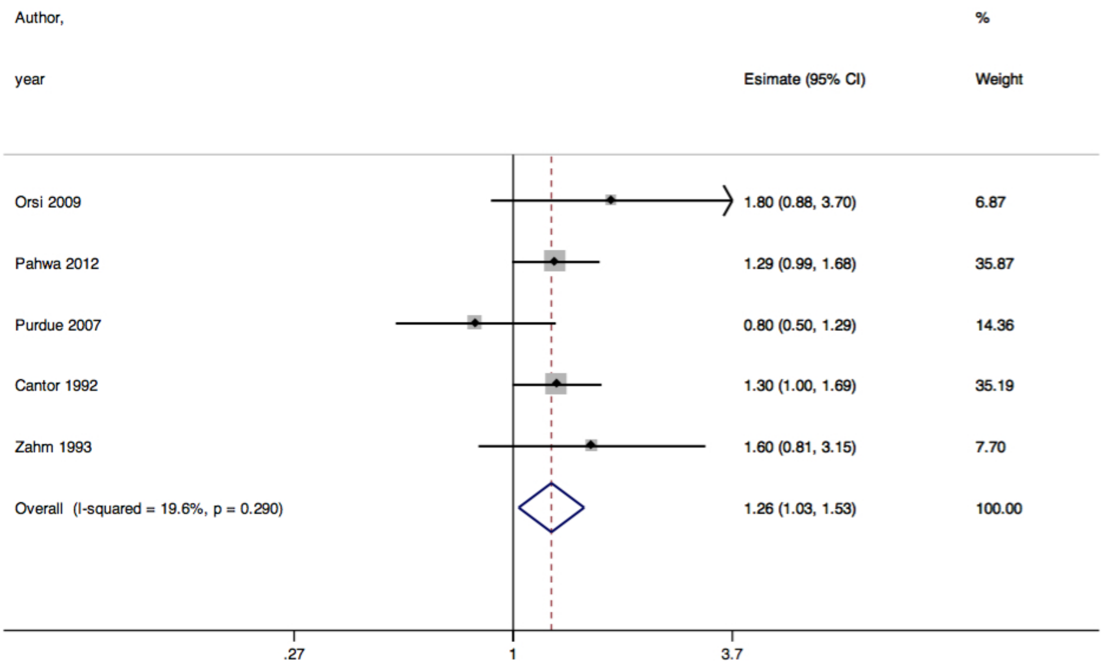

(E)

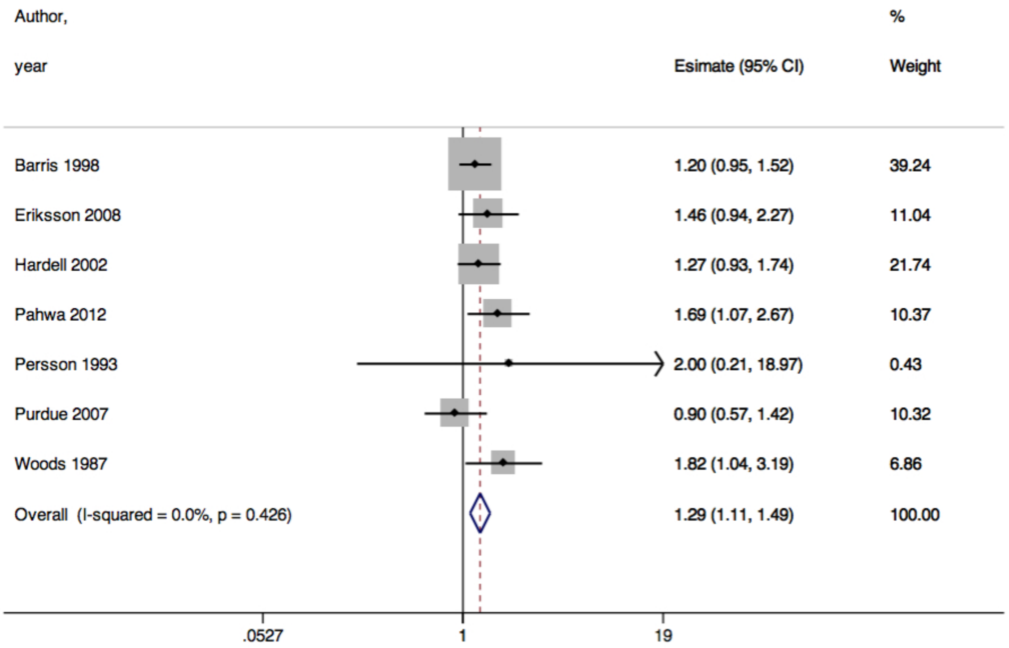

(F)

Notes: 2,4–D, 2,4–Dichlorophenoxyacetic acid; 2,4,5–T, 2,4,5–Trichlorophenoxyacetic acid; DDT, dichlorodiphenyltrichloroethane; MCPA, 2–methyl–4–chlorophenoxyacetic acid.

Table S1. Results of the sensitivity analysis of the effects of gender on the meta– analytic relative risk estimates of association between non–Hodgkin lymphoma and occupational exposure to agricultural pesticides

Chemical	Meta relative risk, 95% CI	I^2	Papers contributing
**Male only population**			
Amide herbicides	1.7, 0.7–3.8	64.0%	[1,2]
Glyphosate	1.7, 1.0–2.9	52.7%	[3–5]
Phenoxy herbicides	1.4, 1.1–1.6	44.1%	[1,2,4,6–8]
2,4–D	1.3, 1.2–1.5	0.0%	[1,6,9]
MCPA	1.5, 0.8–2.7	56.6%	[3,4,6]
Benzoic acid herbicides	1.3, 0.9–1.9	0.0%	[1,2]
Trifluralin	1.0, 0.6–1.5	0.0%	[3,5]
Triazine herbicides	1.5, 0.70–3.4	73.5%	[1,2]
OP insecticides	1.7, 1.3–2.1	39.2%	[6,10]
Diazinon	1.7, 1.2–2.3	0.0%	[5,10]
Malathion	1.8, 1.4–2.2	0.0%	[6,10]
Carbamate insecticides	1.8, 1.3–2.4	0.0%	[5,11]
OC insecticides	1.3, 1.1–1. 6	0.0%	[1,6]
DDT	1.3, 1.1–1.5	27.3%	[4,6,8,12]
Aldrin	1.4, 0.2–11.1	92.0%	[3,5]
Chlordane	1.3, 0.9–1.7	0.0%	[3,5,8]
Lindane	1.9, 1.2–2.9	38.0%	[5,13,14]
**Male and female population**			
Phenoxy herbicides	1.6, 1.0–2.5	42.2%	[15–19]
2,4–D	1.8, 0.5–7.5	88.3%	[19,20]
MCPA	1.6, 0.5–4.8	76.0%	[15,19]
OC insecticides	1.2, 0.5–2.5	70.4%	[16,21]
DDT	1.2, 0.8–1.7	18.0%	[15,18,21]

Notes: 2,4–D, 2,4–Dichlorophenoxyacetic acid; DDT, dichlorodiphenyltrichloroethane; MCPA, 2–methyl–4–chlorophenoxyacetic acid; NHL, OC, Organochlorine; OP; Organophosphorus.

Table S2. Results of the sensitivity analysis of the effects of study design on the meta–analytic relative risk estimates of association between non–Hodgkin lymphoma and occupational exposure to agricultural pesticides, with contributing estimates restricted to case–control studies.

Chemical	Meta relative risk, 95% CI	I^2	Paper contributing
Glyphosate	1.6, 1.1–2.2	36.6%	[3–5,15,16]
Organochlorine insecticides	1.3, 1.1–1.6	0.0%	[1,6,16,22]
Aldrin	1.4, 0.2–11.1	92.0%	[3,5]
Chlordane	1.3, 0.9–1.7	0.0%	[3,5,8]
DDT	1.3, 1.1–1.6	0.0%	[4,6,8,12,15,18]
Lindane	1.9, 1.2–2.9	38.0%	[5,13,14]

Notes: DDT; dichlorodiphenyltrichloroethane.

Table S3. Results of the sensitivity analysis of the effects of diagnosis period on the meta– analytic relative risk estimates of association between non–Hodgkin lymphoma and occupational exposure to agricultural pesticides

Chemical	Meta relative risk, 95% CI	I^2	Papers contributing
**Diagnosis period 1975–1989**			
2,4–D	1.8, 1.0–3.1	76.6%	[1,9,20]
Amide herbicides	1.4, 0.8–2.3	43.2%	[1,2,22]
Glyphosate	2.3, 1.4–4.0	0.0%	[3,4]
MCPA	1.7, 0.7–4.4	63.8%	[3,4]
Phenoxy herbicides	1.4, 1.1–1.7	44.9%	[1,2,4,7,8,17,18,22]
Triazine herbicides	1.4, 0.9–2.2	47.3%	[1,2,23]
Carbamate insecticides	1.6, 1.1–2.4	0.0%	[11,22]
OC insecticides	1.3, 1.0–1.7	0.0%	[1,22]
OP insecticides	1.5, 1.2–1.8	0.0%	[10,22]
Diazinon	1.6, 1.2–2.2	0.0%	[10,20]
Chlordane	1.5, 1.0–2.5	0.0%	[3,8]
Trifluralin	0.9, 0.6–1.3	0.0%	[3,20,22]
Malathion	1.6, 1.3–2.1	0.0%	[10,20]
DDT	1.3, 1.1–1.5	0.0%	[4,8,12,18]
Lindane	2.0, 0.9–4.4	65.0%	[13,14]
**Diagnosis period in the 1990s**			
2,4–D	1.6, 0.8–3.1	79.3%	[6,19,20]
Glyphosate	1. 5, 1.0–2.1	41.1%	[4,5,15,24]
MCPA	1.6, 0.9–2.9	61.9%	[4,6,15,19]
Phenoxy herbicides	1.5, 1.3–1.8	0.5%	[4,6,15,19]
Trifluralin	1.0, 0.6–1.6	0.0%	[5,20]
Aldrin	1.5, 0.2–10.1	90.0%	[5,21]
Chlordane	0.9, 0.6–1.4	42.2%	[5,21]
Diazinon	1.5, 1.0–2.4	0.0%	[5,20]
DDT	1.3, 1.0–1.6	25.4%	[4,6,15,21]
Lindane	1.9, 1.1–3.2	46.6%	[5,14,21]
Malathion	1.9, 1.5–2.5	0.0%	[6,20]
OC insecticides	1.1, 0.7–1.7	66.2%	[6,21]
**Diagnosis period in the 2000s**			
Glyphosate	1.3, 0.9–2.0	31.8%	[15,16,24]
Phenoxy herbicides	1.4, 0.7–3.2	66.7%	[15,16]
Lindane	2.0, 0.8–5.0	70.6%	[14, 21]
OC insecticides	1.2, 0.5–2.5	70.4%	[16,21]

Notes: 2,4–D, 2,4–Dichlorophenoxyacetic acid; DDT, dichlorodiphenyltrichloroethane; MCPA, 2–methyl–4–chlorophenoxyacetic acid; NHL, OC, Organochlorine; OP; Organophosphorus; [1] The first, second, and third editions of the International classification of diseases for oncology were introduced in 1976, 1990, and 2000, respectively.

Table S4. Results of the sensitivity analysis of the effects of geographic region on the meta-analytic relative risk estimates of association between non Hodgkin lymphoma and occupational exposure to agricultural pesticides

Chemical	Meta risk ratio estimate, 95% CI	I^2	Papers contributing
**Only papers that report results from studies conducted in North America**			
Glyphosate	1.3, 1.0–1.8	26.7%	[3,5,24]
Phenoxy herbicides	1.4, 1.1–1.6	12.9%	[1,2,6,8,22]
2,4–D	1.5, 1.1–2.1	66.5%	[1,6,9,20]
MCPA	1.1, 0.7–1.8	0.0%	[3,6]
DDT	1.3, 1.0–1.7	45.1%	[6,8,12,21]
OC insecticides	1.2, 1.0–1.5	24.7%	[1,6,21,22]
OP insecticides	1.6, 1.3–2.0	15.1%	[6,10,22]
Lindane	1.5, 1.2–1.9	0.0%	[5,13,21]
**Only papers that report results from studies conducted in the United States**			
2,4–D	1.8, 1.0–3.1	76.6%	[1,6,9,20]
Amide herbicides	1.4, 0.8–2.3	43.2%	[1,2,22]
Glyphosate	1.5, 0.8–2.8	58.5%	[3,24]
Phenoxy herbicides	1.3, 1.0–1.7	27.1%	[1,2,8,22]
Trifluralin	0.9, 0.6–1.3	0.0%	[3,20,22]
Triazine herbicides	1.4, 0.9–2.2	47.3%	[1,2,22]
Aldrin	0.5, 0.4–0.8	0.0%	[3,21]
Carbamate insecticides	1.6, 1.1–2.4	0.0%	[22,23]
Chlordane	1.1, 0.7–2.0	55.0%	[3,8,21]
DDT	1.2, 0.9–1.7	44.8%	[8,12,21]

Table S4. *Cont.*

Chemical	Meta risk ratio estimate, 95% CI	I^2	Papers contributing
**Only papers that report results from studies conducted in the United States**			
Diazinon	1.6, 1.2–2.2	0.0%	[10,20]
Lindane	1.4, 1.1–1.9	0.0%	[13,21]
Malathion	1.6, 1.3–2.1	0.0%	[10,20]
OC insecticides	1.2, 0.8–1.7	47.5%	[1,21,22]
OP insecticides	1.5, 1.2–1.8	0.0%	[10,22]
**Only papers that report results from studies conducted in European countries**			
Glyphosate	1.7, 1.0–3.1	42.8%	[4,15,16]
Phenoxy herbicides	1.6, 1.2–2.1	29.1%	[4,15–19]
MCPA	1.9, 0.9–3.8	64.8%	[4,15,19]
**Only papers that report results from studies conducted in Sweden**			
Glyphosate	2.2, 1.3–3.8	0.0%	[4,15]
MCPA	2.7, 1.6–4.4	0.0%	[4,15]
Phenoxy herbicides	1.9, 1.4–2.4	0.0%	[4,15,17,18]
DDT	1.3, 1.0–1.7	0.0%	[4,15,18]

Notes: 2,4–D, 2,4–Dichlorophenoxyacetic acid; DDT, dichlorodiphenyltrichloroethane; MCPA, 2–methyl–4–chlorophenoxyacetic acid; NHL, OC, Organochlorine; OP; Organophosphorus

Table S5. Results of the sensitivity analysis of the effects of control source on the meta– analytic relative risk estimates of association between non–Hodgkin lymphoma and occupational exposure to agricultural pesticides, with contributing estimates restricted to those from population–based case–control studies.

Chemical	Meta risk ratio estimate, 95% CI	I^2	Papers contributing
HERBICIDES			
Amide herbicides	1.4, 0.8–2.3	43.2%	[1,2,22]
Glyphosate	1.7, 1.2–2.6	39.0%	[3,4,5,15]
Phenoxy herbicides	1.5, 1.2–1.7	20.7%	[1,2,4,6,8,15,17–19,22]
Triazine herbicides	1.4, 0.9–2.2	47.3%	[1,2,22]
**INSECTICIDES**			
Organochlorine insecticides	1.2, 1.0–1.5	24.7%	[1,6,21,22]
Organophosphate insecticides	1.6, 1.4–1.8	0.0%	[1,6,10,22]

Table S6. Results of the sensitivity analysis of the effects of paper contributing on the meta– analytic relative risk estimates of association between non–Hodgkin lymphoma and occupational exposure to agricultural pesticides.

Chemical	Meta estimate, 95% CI	I^2	Change	Papers contributing
HERBICIDES				
Alachlor	0.9, 0.6–1.5	69.7%	Use Cantor 1992 [1] instead of De Roos 2003 [3]	[1,25]
Glyphosate	1.3, 1.0–1.7	18.2%	Use Cantor 1992 [1] instead of De Roos 2003 [3]	[1,24]
2,4–D	1.3, 0.8–2.1	82.5%	Use De Roos 2003 [3] instead of Cantor 1992 [1] and Zahm 1990 [9]	[3,6,19,20]
Carbamate herbicides	1.2, 0.5–2.6	24.8%	Use Cantor 1992 [1] and Hoar 1986 [2] instead of Zheng 2001 [11]	[1,2,16,22]
Trifluralin	1.1, 0.7–1.8	40.0%	Use Cantor 1992 [1] and Hoar 1986 [2] instead of De Roos 2003[3]	[1,2,5,20,22]
INSECTICIDES				
OP insecticides	1.7, 1.4–2.0	0.0%	Use Cantor 1992 [1] instead of Waddell 2001 [10]	[1,6,16,22]
Diazinon	1.5, 1.1–2.1	0.0%	Use Cantor 1992 [1] instead of Waddell 2001 [10]	[1,3,5,20]
Diazinon	1.7, 1.2–2.4	0.0%	Use De Roos 2003 [3] instead of Cantor 1992 [1] and instead of Waddell 2001 [10]	[3,5,20]
Dimethoate	1.2, 0.7–2.0	0.0%	Use De Roos 2003 [3] instead of Waddell 2001 [10]	[3,5]
Malathion	1.7, 1.3–2.2	13.5%	Use Cantor 1992 [1] (use of malathion on animals) instead of Waddell 2001 [10] or De Roos 2003 [3]	[1,6,20]
Malathion	1.8, 1.4–2.4	0.0%	Use Cantor 1992 [1] (use of malathion on crops) instead of Waddell 2001 [10] or De Roos 2003 [3]	[1,6,20]
Malathion	1.6, 1.2–2.3	37.2%	Use De Roos 2003 [3] instead of Waddell 2001 [10] and Cantor 1992 [1]	[3,6,20]
Carbaryl	1.9, 1.3–2.9	0.0%	Use Cantor 1992 [1] instead of Zheng 2001 [11]	[1,5]
Carbaryl	1.5, 0.7–3.1	64.7%	Use De Roos 2003 [3] instead of Cantor 1992 [1] or Zheng 2001 [11]	[3,5]
Carbofuran	1.1, 0.7–1.8	0.0%	Use Cantor 1992 [1] instead of Zheng 2001 [11]	[1,5]

Table S6. *Cont.*

Chemical	Meta estimate, 95% CI	I^2	Change	Papers contributing
Carbofuran	1.1, 0.6–2.0	23.0%	Use De Roos 2003 [3] instead of Cantor 1992 [1] or Zheng 2001 [11]	[3,5]
DDT	1.3, 1.1–1. 5	0.0%	Use Cantor 1992 [1] (use of DDT on animals) instead of Baris 1998 [12]	[1,4,6,8,15,18,21]
DDT	1.3, 1.2–1. 6	9.1%	Use Cantor 1992 [1] (use of DDT on crops) instead of Baris 1998 [12]	[1,4,6,8,15,18,21]
Methoxychlor	1.0, 0.8–1.4	0.0%	Use Cantor 1992 [1] instead of De Roos 2003 [3]	[1,5]
Aldrin	1.3, 0.5–2.9	80.2%	Use Cantor 1992 [1] instead of De Roos 2003 [3]	[1,5,21]
Chlordane	1.2, 0.8–1.7	48.7%	Use Cantor 1992 [1] (Use of chlordane on animals) instead of De Roos 2003 [3]	[1,5,8,21]
Chlordane	1.1, 0.8–1.7	42.1%	Use Cantor 1992 [1] (Use of chlordane on crops) instead of De Roos 2003 [3]	[1,5,8,21]
Dieldrin	1.0, 0.4–2.2	50.8%	Use Cantor 1992 [1] instead of De Roos 2003[3]	[1,21]
Heptachlor	1.0, 0.7–1.7	20.5%	Use Cantor 1992 [1] instead of De Roos 2003[3]	[1,21]
Lindane	1.62, 1.16–2.27	30.6%	Use Cantor 1992 [1] (use of lindane on animals) instead of Blair 1998 [13] and De Roos 2003 [3]	[1,5,14,21]
Lindane	1.85, 1.27–2.69	23.30 %	Use Cantor 1992 [1] (use of lindane on crops) instead of Blair 1998 [13]and De Roos 2003 [3]	[1,5,14,21]
Lindane	1.62, 1.08–2.41	39.20 %	Use De Roos 2003 [3] instead of Cantor 1992 [1] or Blair 1998 [13]	[3,5,14,21]
Toxaphene	1.25, 0.72–2.19	23.50 %	Use Cantor 1992 [1] (use of toxaphene on animals) instead of De Roos 2003 [3]	[1,20,21]
Toxaphene	1.50, 0.96–2.33	0.00%	Use Cantor 1992 [1] (use of toxaphene on crops) instead of De Roos 2003 [3]	[1,20,21]

Notes: 2,4–D, 2,4–Dichlorophenoxyacetic acid; DDT, dichlorodiphenyltrichloroethane; MCPA, 2–methyl–4–chlorophenoxyacetic acid; NHL, OC, Organochlorine; OP; Organophosphorus.

References

1. Cantor, K.P.; Blair, A.; Everett, G.; Gibson, R.; Burmeister, L.F.; Brown, L.M.; Schuman, L.; Dick, F.R. Pesticides and other agricultural risk factors for non-Hodgkin's lymphoma among men in Iowa and Minnesota. *Cancer Res.* **1992**, *52*, 2447–2455.
2. Hoar, S.K.; Blair, A.; Holmes, F.F.; Boysen, C.D.; Robel, R.J.; Hoover, R.; Fraumeni, J.F., Jr. Agricultural herbicide use and risk of lymphoma and soft-tissue sarcoma. *JAMA* **1986**, 256, 1141–1147.
3. De Roos, A.J.; Zahm, S.H.; Cantor, K.P.; Weisenburger, D.D.; Holmes, F.F.; Burmeister, L.F.; Blair, A. Integrative assessment of multiple pesticides as risk factors for non-Hodgkin's lymphoma among men. *Occup. Environ. Med.* **2003**, *60*, doi:10.1136/oem.60.9.e11.
4. Hardell, L.; Eriksson, M.; Nordstrom, M. Exposure to pesticides as risk factor for non-Hodgkin's lymphoma and hairy cell leukemia: Pooled analysis of two Swedish case-control studies. *Leuk. Lymphoma* **2002**, *43*, 1043–1049.
5. McDuffie, H.H.; Pahwa, P.; McLaughlin, J.R.; Spinelli, J.J.; Fincham, S.; Dosman, J.A.; Robson, D.; Skinnider, L.F.; Choi, N.W. Non-Hodgkin's lymphoma and specific pesticide exposures in men: Cross-Canada study of pesticides and health. *Cancer Epidemiol. Biomark. Prev.* **2001**, *10*, 1155–1163.
6. Pahwa, M.; Harris, S.A.; Hohenadel, K.; McLaughlin, J.R.; Spinelli, J.J.; Pahwa, P.; Dosman, J.A.; Blair, A. Pesticide use, immunologic conditions, and risk of non-Hodgkin lymphoma in Canadian men in six provinces. *Int. J. Cancer* **2012**, *131*, 2650–2659.
7. Pearce, N.E.; Sheppard, R.A.; Smith, A.H.; Teague, C.A. Non-Hodgkin's lymphoma and farming: An expanded case-control study. *Int. J. Cancer* **1987**, *39*, 155–161.
8. Woods, J.S.; Polissar, L.; Severson, R.K.; Heuser, L.S.; Kulander, B.G. Soft tissue sarcoma and non-Hodgkin's lymphoma in relation to phenoxyherbicide and chlorinated phenol exposure in western Washington. *J. Natl. Cancer Inst.* **1987**, *78*, 899–910
9. Zahm, S.H.; Weisenburger, D.D.; Babbitt, P.A.; Saal, R.C.; Vaught, J.B.; Cantor, K.P.; Blair, A. A case-control study of non-Hodgkin's lymphoma and the herbicide 2,4-dichlorophenoxyacetic acid (2,4-D) in eastern Nebraska. *Epidemiology* **1990**, *1*, 349–356.
10. Waddell, B.L.; Zahm, S.H.; Baris, D.; Weisenburger, D.D.; Holmes, F.; Burmeister, L.F.; Cantor, K.P.; Blair, A. Agricultural use of organophosphate pesticides and the risk of non-Hodgkin's lymphoma among male farmers (United States). *Cancer Cause. Control* **2001**, *12*, 509–517.
11. Zheng, T.; Zahm, S.H.; Cantor, K.P.; Weisenburger, D.D.; Zhang, Y.; Blair, A. Agricultural exposure to carbamate pesticides and risk of non-Hodgkin lymphoma. *J. Occup. Environ. Med.* **2001**, *43*, 641–649.
12. Baris, D.; Zahm, S.H.; Cantor, K.P.; Blair, A. Agricultural use of DDT and risk of non-Hodgkin's lymphoma: Pooled analysis of three case-control studies in the United States. *Occup. Environ. Med.* **1998**, *55*, 522–527.
13. Blair, A.; Cantor, K.P.; Zahm, S.H. Non-hodgkin's lymphoma and agricultural use of the insecticide lindane. *Amer. J. Ind. Med.* **1998**, *33*, 82–87.
14. Rafnsson, V. Risk of non-Hodgkin's lymphoma and exposure to hexachlorocyclohexane, a nested case-control study. *Eur. J. Cancer* **2006**, *42*, 2781–2785.

15. Eriksson, M.; Hardell, L.; Carlberg, M.; Akerman, M. Pesticide exposure as risk factor for non-Hodgkin lymphoma including histopathological subgroup analysis. *Int. J. Cancer* **2008**, *123*, 1657–1663.
16. Orsi, L.; Delabre, L.; Monnereau, A.; Delval, P.; Berthou, C.; Fenaux, P.; Marit, G.; Soubeyran, P.; Huguet, F.; Milpied, N.; *et al*. Occupational exposure to pesticides and lymphoid neoplasms among men: Results of a French case-control study. *Occup. Environ. Med.* **2009**, *66*, 291–298.
17. Persson, B.; Dahlander, A.M.; Fredriksson, M.; Brage, H.N.; Ohlson, C.G.; Axelson, O. Malignant lymphomas and occupational exposures. *Brit. J. Ind. Med.* **1989**, *46*, 516–520.
18. Persson, B.; Fredriksson, M.; Olsen, K.; Boeryd, B.; Axelson, O. Some occupational exposures as risk factors for malignant lymphomas. *Cancer* **1993**, *72*, 1773–1778.
19. Miligi, L.; Costantini, A.S.; Veraldi, A.; Benvenuti, A.; Vineis, P. Cancer and pesticides: An overview and some results of the Italian Multicenter case-control study on hematolymphopoietic malignancies. In *Living in a Chemical World: Framing the Future in Light of the Past*; Blackwell Publishing: Oxford, UK, 2006; Volume 1076, pp. 366–377.
20. Mills, P.K.; Yang, R.; Riordan, D. Lymphohematopoietic cancers in the United Farm Workers of America (UFW), 1988–2001. *Cancer Cause. Control* **2005**, *16*, 823–830.
21. Purdue, M.P.; Hoppin, J.A.; Blair, A.; Dosemeci, M.; Alavanja, M.C. Occupational exposure to organochlorine insecticides and cancer incidence in the Agricultural Health Study. *Int. J. Cancer* **2007**, *120*, 642–649.
22. Zahm, S.H.; Weisenburger, D.D.; Saal, R.C.; Vaught, J.B.; Babbitt, P.A.; Blair, A. The role of agricultural pesticide use in the development of non-Hodgkin's lymphoma in women. *Arch. Environ. Health* **1993**, *48*, 353–358.
23. Zahm, S.H.; Weisenburger, D.D.; Cantor, K.P.; Holmes, F.F.; Blair, A. Role of the herbicide atrazine in the development of non-Hodgkin's lymphoma. *Scand. J. Work Environ. Health* **1993**, *19*, 108–114.
24. De Roos, A.J.; Blair, A.; Rusiecki, J.A.; Hoppin, J.A.; Svec, M.; Dosemeci, M.; Sandler, D.P.; Alavanja, M.C. Cancer incidence among glyphosate-exposed pesticide applicators in the Agricultural Health Study. *Environ. Health Perspect.* **2005**, *113*, 49–54.
25. Lee, W.J.; Hoppin, J.A.; Blair, A.; Lubin, J.H.; Dosemeci, M.; Sandler, D.P.; Alavanja, M.C. Cancer incidence among pesticide applicators exposed to alachlor in the agricultural health study. *Amer. J. Epidemiol.* **2004**, *159*, 373–380.

© 2014 by the authors; licensee MDPI, Basel, Switzerland. This article is an open access article distributed under the terms and conditions of the Creative Commons Attribution license (http://creativecommons.org/licenses/by/3.0/).
